# Copper-catalyzed enantioselective conjugate reduction of α,β-unsaturated esters with chiral phenol–carbene ligands

**DOI:** 10.3762/bjoc.16.50

**Published:** 2020-03-31

**Authors:** Shohei Mimura, Sho Mizushima, Yohei Shimizu, Masaya Sawamura

**Affiliations:** 1 Department of Chemistry, Faculty of Science, Hokkaido University, Kita 10 Nishi 8, Kita-ku, Sapporo, Hokkaido 060-0810, Japan; 2 Institute for Chemical Reaction Design and Discovery (WPI-ICReDD), Hokkaido University, Kita 21 Nishi 10, Kita-ku, Sapporo, Hokkaido 001-0021, Japan

**Keywords:** catalyst, chiral NHC, conjugate reduction, copper catalysis, enantioselective reaction

## Abstract

A chiral phenol–NHC ligand enabled the copper-catalyzed enantioselective conjugate reduction of α,β-unsaturated esters. The phenol moiety of the chiral NHC ligand played a critical role in producing the enantiomerically enriched products. The catalyst worked well for various (*Z*)-isomer substrates. Opposite enantiomers were obtained from (*Z*)- and (*E*)-isomers, with a higher enantiomeric excess from the (*Z*)-isomer.

## Introduction

Since the leading work of Stryker and co-workers on triphenylphosphine-stabilized copper hydride complexes [1–2], copper hydrides have been widely used for conjugate reductions of α,β-unsaturated carbonyl compounds [3]. Especially a chiral copper catalyst combined with a stoichiometric amount of a silane reagent, which generated copper hydride in situ, has successfully been utilized for enantioselective reactions with β,β-disubstituted α,β-unsaturated carbonyl compounds [4–11]. The pioneering work of Buchwald and co-workers on the enantioselective conjugate reduction of α,β-unsaturated esters using a chiral *p*-tol-BINAP/copper catalyst established the excellent utility of chiral bisphosphine ligands for this type of reaction [4]. Surprisingly, however, chiral ligands based on *N*-heterocyclic carbenes (NHCs) [12] have not been applied to the conjugate reduction of α,β-unsaturated carbonyl compounds, while an achiral NHC/copper catalyst has successfully been utilized in this reaction [13].

Meanwhile, we devoted our effort to develop novel enantioselective C–C bond formation reactions utilizing chiral phenol–NHC/copper catalyst systems [14–18], in which the phenol group of the NHC ligand plays crucial roles in both the catalytic activity and stereoselectivity [19–21]. Notably, these catalyst systems were also applicable for three-component coupling reactions using hydrosilanes as hydride reagents [17]. Based on this knowledge, we decided to investigate the effects of the phenol–NHC ligand on the copper-catalyzed enantioselective conjugate reduction of α,β-unsaturated esters with hydrosilanes, placing a focus on (*Z*)-isomer substrates, which generally gave slightly lower enantiomeric excess with the chiral bisphosphines compared to the (*E*)-isomer substrates.

## Results and Discussion

### Optimization

The initial investigation of the reaction conditions was carried out with ethyl (*Z*)-3-phenylbut-2-enoate (**1a**) as a substrate (Table 1). When chiral NHC precursor **L1**·HBF_4_ (10 mol %) was used in combination with CuCl (10 mol %) and LiO*t*-Bu (20 mol %) for the conjugate reduction of **1a** with diethoxymethylsilane (4 equiv) as a reductant and *t*-AmOH (1 equiv) as a protonation reagent in DMA as the solvent at 25 °C for 15 h, the product **2a** was produced in 98% yield (^1^H NMR analysis) with a promising enantioselectivity of 69% ee (Table 1, entry 1). When the phenolic hydroxy group of **L1** was changed to a methoxy group (in **L2**), the enantioselectivity drastically dropped to −10% ee, while the yield remained 99% (Table 1, entry 2). Similarly, *N*,*N'*-dimesityl-NHC **L3**, which lacked an oxygen functionality in the *N*-aryl group, showed poor enantioselectivity (9% ee) with high yield (99% yield, Table 1, entry 3). Thus, the hydroxy group of **L1** was essential for the enantioselectivity by the catalyst. When the mesityl group of **L1** was changed to a bulkier 2-Me-4,6-Cy_2_-C_6_H_2_ group in **L4**, the enantioselectivity was markedly improved to 90% ee, with a high yield (97%, Table 1, entry 4). A naphthol substituent on the nitrogen atom of the NHC (in **L5** and **L6**) instead of the phenol substituent was not suitable, giving significantly lower enantioselectivities (Table 1, entry 5, 99% yield, 26% ee; entry 6, 97% yield, 58% ee).

**Table 1:** Optimization of the copper-catalyzed enantioselective conjugate reduction of **1a**.^a^

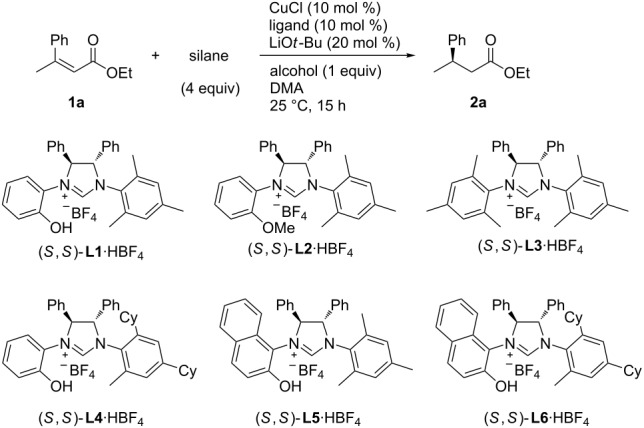					

entry	ligand	silane	alcohol	yield (%)	ee (%)

1	(*S*,*S*)-**L1**·HBF_4_	(EtO)_2_MeSiH	*t*-AmOH	98	69
2	(*S*,*S*)-**L2**·HBF_4_	(EtO)_2_MeSiH	*t*-AmOH	99	−10
3	(*S*,*S*)-**L3**·HBF_4_	(EtO)_2_MeSiH	*t*-AmOH	99	9
4	(*S*,*S*)-**L4**·HBF_4_	(EtO)_2_MeSiH	*t*-AmOH	97	90
5	(*S*,*S*)-**L5**·HBF_4_	(EtO)_2_MeSiH	*t*-AmOH	99	26
6	(*S*,*S*)-**L6**·HBF_4_	(EtO)_2_MeSiH	*t*-AmOH	97	58
7	(*S*,*S*)-**L4**·HBF_4_	(MeO)_2_MeSiH	*t*-AmOH	98	84
8	(*S*,*S*)-**L4**·HBF_4_	(TMSO)_2_MeSiH	*t*-AmOH	94	71
9	(*S*,*S*)-**L4**·HBF_4_	(EtO)_3_SiH	*t*-AmOH	0	–
10	(*S*,*S*)-**L4**·HBF_4_	PMHS	*t*-AmOH	5	–
11	(*S*,*S*)-**L4**·HBF_4_	(EtO)_2_MeSiH	*t*-BuOH	99	82
12	(*S*,*S*)-**L4**·HBF_4_	(EtO)_2_MeSiH	iPrOH	7	–
13	(*S*,*S*)-**L4**·HBF_4_	(EtO)_2_MeSiH	MeOH	4	–

^a^The yield was determined by ^1^H NMR analysis using 1,1,2,2-tetrachloroethane as an internal standard. The enantiomeric excess (ee) was determined by HPLC analysis with a chiral stationary phase column CHIRALCEL^®^ OD-H.

Further optimization of the conditions was conducted with **L4**. Changing the silane group affected both the reactivity and selectivity (Table 1, entries 7–10), while the replacement of the ethoxy groups of (EtO)_2_MeSiH with methoxy or trimethylsilyloxy groups resulted in only moderate reductions in the enantioselectivity and high yields. At the same time, trialkoxysilane (EtO)_3_SiH and polymeric silane PMHS gave only trace amounts of the product.

The nature of the alcoholic protonation reagent also had a strong impact. The presence of a tertiary alcohol, *t*-AmOH or *t*-BuOH, was essential for the reaction to occur with a reasonable yield, while iPrOH and MeOH markedly suppressed the reaction (Table 1, entries 12 and 13). However, the bulkier *t*-AmOH was superior to *t*-BuOH in terms of enantioselectivity (Table 1, entries 4 and 11).

### Substrate scope

Having established optimized conditions for the reaction of **1a** (**2a**, 97% yield, 90% ee (*R*), Table 2, entry 1), the scope of α,β-unsaturated carbonyl compounds was examined. Because the separation of the product from silicon-based byproducts was troublesome, the isolated yields were lower than the yields determined by NMR spectroscopy (**2a**, 52%, Table 2, entry 1).

**Table 2:** Substrate scope of the copper-catalyzed enantioselective conjugate reduction.^a^

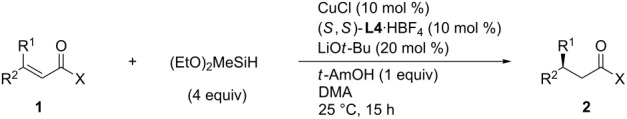				

entry	substrate	product	yield (%)	ee (%)

1	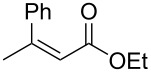 **1a**	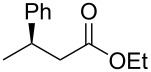 **2a**	97 (52)	90
2	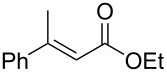 (*E*)-**1a**	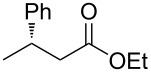 (*S*)-**2a**	>99 (75)	82
3^b^	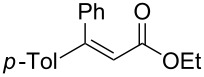 **1b**	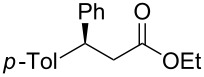 **2b**	>99 (60)	75
4	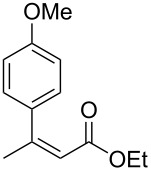 **1c**	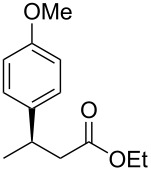 **2c**	>99 (44)	84
5	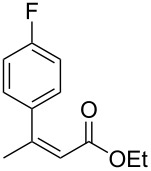 **1d**	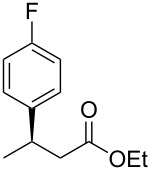 **2d**	96 (74)	76
6^b,c^	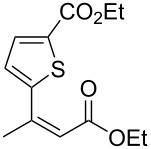 **1e**	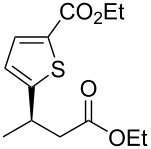 **2e**	>99 (75)	84
7	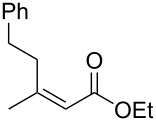 **1f**	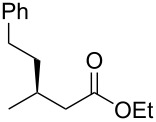 **2f**	>99 (45)	85
8^b,c^	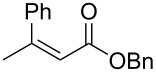 **1g**	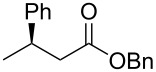 **2g**	>99 (55)	70
9	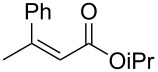 **1h**	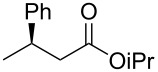 **2h**	>99 (79)	83
10	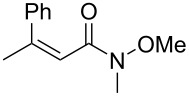 **1i**	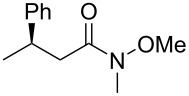 **2i**	81 (59)	79

^a^The yields were determined by ^1^H NMR analysis using 1,1,2,2-tetrachloroethane as an internal standard. Isolated yields are shown in parentheses. ^b^*t*-BuOH was used instead of *t*-AmOH. ^c^(EtO)_2_MeSiH (1 equiv) was used.

When (*E*)-**1a** was used as the substrate, the opposite enantiomer (*S*)-**2a** was obtained in >99% yield, with slightly lower enantioselectivity (Table 2, entry 2, 82% ee (*S*) vs 90% ee (*R*) in entry 1). The inversion of the absolute configuration of the product depended on the *E*/*Z* geometry of the substrates, and this was analogous to the reported results obtained with the chiral bisphosphine ligand systems, while the observation of a higher enantioselectivity for the (*Z*)-isomer substrate **1a** was characteristic for the phenol–NHC chiral ligand [4,6,8]. The result suggested that the chiral catalyst may mainly discriminate the hydrogen atom and the ethoxycarbonyl group at the α-position rather than the two substituents at the β-position. In good agreement with this assumption is the reaction of substrate **1b**, carrying a phenyl group and a *p*-tolyl group as β-substituents, which seemed to be difficult for the catalyst to differentiate, both sterically and electronically, affording the product **2b** in good enantioselectivity (Table 2, entry 3, >99% yield, 75% ee).

Next, the effects of the β-substituent (R^1^) were examined. Both electron-donating and -withdrawing substituents on the *para*-position of the β-aryl substituent gave excellent reactivities and good enantioselectivities (Table 2, entry 4, **2c**: >99% yield, 84% ee; entry 5, **2d**: 96% yield, 76% ee). Further, a heteroaryl substituent, 2-ethoxycarbonylthiophene, was tolerated (Table 2, entry 6, **2e**: >99% yield, 84% ee), and a β-alkyl-substituted substrate **1f** was also competent (entry 7, **2f**: >99% yield, 85% ee).

The structure of the ester moiety affected the stereoselectivities: Lower enantioselectivities were observed with benzyl ester **2g** (Table 2, entry 8, >99% yield, 70% ee) and isopropyl ester **2h** (Table 2, entry 9, >99% yield, 83% ee). In addition to the α,β-unsaturated esters, an α,β-unsaturated Weinreb amide **1i** afforded the corresponding product **2i** with comparable results (Table 2, entry 10, 81% yield, 79% ee).

### Proposed catalytic cycle

**Scheme 1:**
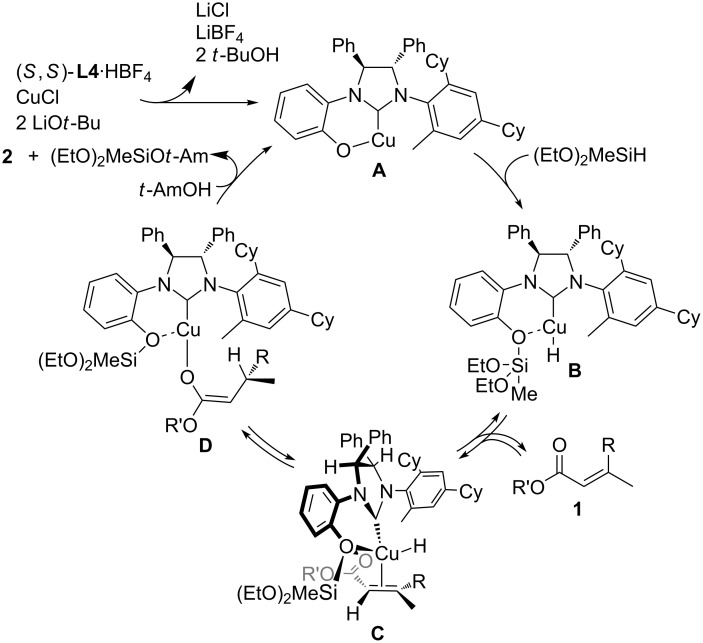
Proposed catalytic cycle.

Based on the experimental observations and previous knowledge of the catalysis of phenol–NHC chiral ligands [14–18], we propose a catalytic cycle as shown in Scheme 1. LiO*t*-Bu abstracts the two protons of the acidic imidazolium C–H and phenol O–H groups of the **L4**·HBF_4_ adduct in the presence of CuCl to generate a phenoxy copper(I) species **A**. As a result, all LiO*t*-Bu (20 mol %) is consumed in this step. Thus, the system is neutral. Transmetalation between **A** and (EtO)_2_MeSiH produces the copper hydride species **B**. This transmetalation adds the silyl group to the phenoxy oxygen atom of the NHC ligand. The coordination of an α,β-unsaturated carbonyl compound **1** to the copper atom occurs in such a way that the bulky *O*-silyl group of the copper catalyst can avoid steric repulsions with the alkoxycarbonyl group of the substrate **1**, forming π-complex **C**, and thus explaining the marked influence of the hydrosilane structure on the enantioselectivity. During this stereodetermining step, coordination of the phenoxy oxygen atom to the copper atom may render the chiral environment better defined. Then, the π-complex **C** undergoes 1,4-hydrocupration to afford copper enolate **D**. During our initial investigations, we observed configurational isomerization from **1a** to (*E*)-**1a** when the reaction was conducted in the absence of alcoholic protonation reagents. This observation implied that the 1,4-hydrocupration step (**C** to **D**) is reversible. Finally, protonation of **D** by *t*-AmOH gives the product **2** and silyl ether (EtO)_2_MeSiO*t*-Am, regenerating the phenoxy copper(I) complex **A**. Due to the reversibility of the 1,4-hydrocupration, the choice of the alcoholic protonation reagent affects both the reactivity and enantioselectivity.

## Conclusion

A chiral phenol–NHC ligand efficiently promoted the enantioselective conjugate reduction of α,β-unsaturated esters with a hydrosilane. To the best of our knowledge, this is the first demonstration of the applicability of chiral NHC ligands in Cu-catalyzed enantioselective conjugate reductions. The phenolic *N*-substituent of the chiral NHC ligand was essential for the high enantioselectivity. Good enantioselectivities were observed for the various (*Z*)-configured substrates with different β-substituents. Further investigations on the catalyst development based on this knowledge are ongoing in our laboratory.

## Experimental

**A typical procedure for the copper-catalyzed enantioselective conjugate reduction** (Table 1, entry 4; Table 2, entry 1): In a glove box, CuCl (1.5 mg, 0.015 mmol), **L4**⋅HBF_4_ (9.8 mg, 0.015 mmol), and LiO*t*-Bu (2.4 mg, 0.03 mmol) were placed in a vial containing a magnetic stirring bar. The vial was sealed with a teflon-coated silicon rubber septum. *N*,*N*-Dimethylacetamide (0.90 mL) was added to the vial, and then the mixture was stirred at room temperature for 5 min. Next, *t*-AmOH (16.3 μL, 0.15 mmol) was added, and the vial was taken out of the glove box. To the reaction mixture was added **1a** (28.5 mg, 0.15 mmol). After stirring for 5 min at room temperature, diethoxymethylsilane (101 μL, 0.60 mmol) was added to the mixture. After stirring for 15 h at 25 °C, the reaction mixture was diluted with diethyl ether (1.0 mL) and quenched with H_2_O (0.6 mL). The organic layer was separated, and the aqueous layer was extracted three times with diethyl ether. The combined organic layer was filtered through a short plug of silica gel by using diethyl ether as an eluent. After the solvent was removed under reduced pressure, the yield of the product was determined to be 97% by ^1^H NMR analysis with 1,1,2,2-tetrachloroethane as an internal standard. After a rough purification of the crude product by silica gel chromatography (eluent: 0 to 1% EtOAc/hexane), the collected residue was further purified by GPC (eluent: CHCl_3_) to afford the pure product **2a** as colorless oil (15.1 mg, 52% yield, 90% ee). ^1^H NMR (400 MHz, CDCl_3_) δ 1.17 (t, *J* = 7.2 Hz, 3H), 1.29 (d, *J* = 7.2 Hz, 3H), 2.50–2.63 (m, 2H), 3.27 (sext, *J* = 7.2 Hz, 1H), 4.06 (q, *J* = 7.2 Hz, 2H), 7.16–7.22 (m, 3H), 7.28 (t, *J* = 7.2 Hz, 2H); ^13^C NMR (100.5 Hz, CDCl_3_) δ 14.1, 21.7, 36.4, 42.9, 60.2, 126.3, 126.7, 128.4, 145.6, 172.3; [α]_D_^27.3^ −23.2 (*c* 1.25, CHCl_3_). The spectral data matched those reported in the literature [22].

The ee value was determined by HPLC analysis with a chiral stationary column CHIRALCEL^®^ OD-H (Daicel Chemical Industries, 4.6 mm, 250 mm, hexane/2-propanol, 98:2, v/v, 0.5 mL/min, 40 °C, 220 nm UV detector, retention time = 9.1 min for the (*R*)-isomer and 13.3 min for the (*S*)-isomer). The absolute configuration of **2a** was assigned by the comparison of the optical rotation with the same compound prepared by a reported method [4].

## Supporting Information

File 1 Experimental procedures, characterization data, HPLC charts, and NMR spectra (^1^H, ^13^C) for the new compounds.
