# Supplementary material for: Copper-catalyzed enantioselective conjugate reduction of α,β-unsaturated esters with chiral phenol–carbene ligands
Source: Beilstein J Org Chem. 2020 Mar 31;16:537–43. doi: 10.3762/bjoc.16.50 (PMC7136546; doi:10.3762/bjoc.16.50)
Supplement: File 1 — Experimental procedures, characterization data, HPLC charts, and NMR spectra (1H, 13C) for the new compounds. [file Beilstein_J_Org_Chem-16-537-s001.pdf]

BEILSTEIN JOURNAL OF ORGANIC CHEMISTRY

Supporting Information

for

Copper-catalyzed enantioselective conjugate reduction of α,β-unsaturated esters with chiral phenol–carbene ligands

Shohei Mimura, Sho Mizushima, Yohei Shimizu and Masaya Sawamura

Beilstein J. Org. Chem. **2020,** *16,* 537–543. doi:10.3762/bjoc.16.50

Experimental procedures, characterization data, HPLC charts, and NMR spectra (^{1}H, ^{13}C) for the new compounds

License and Terms: This is a supporting information file under the terms of the Creative Commons Attribution License (http://creativecommons.org/licenses/by/4.0). Please note that the reuse, redistribution and reproduction in particular requires that the authors and source are credited.
The license is subject to the *Beilstein Journal of Organic Chemistry* terms and conditions: (https://www.beilstein-journals.org/bjoc)

Table of Contents

Instrumentation and Chemicals	S1
Solvent Screening	S2
Preparation of α,β-Unsaturated Esters	S3–S6
Characterization Data for α,β-Unsaturated Esters	S6–S8
Procedure for the Copper-catalyzed Enantioselective Conjugate Reduction	S8
Characterization Data for Reduction Products	S8–S12
References	S13
HPLC Charts	S14–S23
NMR Spectra	S24–S37

Instrumentation and Chemicals

NMR spectra were recorded on a JEOL ECA-400, operating at 400 MHz for ^{1}H NMR and 100.5 MHz for ^{13}C NMR. Chemical shift values for ^{1}H and ^{13}C are referenced to Me_4Si and the residual solvent resonances, respectively. Mass spectra were obtained with Thermo Fisher Scientific Exactive, JEOL JMS-T100GCV at the Instrumental Analysis Division, Global Facility Center, Creative Research Institution, Hokkaido University. Chemical shifts are reported in δ ppm. HPLC analyses were conducted on a HITACHI ELITE LaChrom system with a HITACHI L-2455 diode array detector or a Shimadzu HPLC systems (system controller: CBM-20A, pump: LC-20AR; detector: SPD-20A). TLC analyses were performed on commercial glass plates bearing a 0.25 mm layer of Merck silica gel 60 F_{254}. Silica gel (Kanto Chemical Co., Silica gel 60 N, spherical, neutral) was used for column chromatography.

All reactions were carried out under nitrogen or argon atmosphere. Materials were obtained from commercial suppliers or prepared according to standard procedures unless otherwise noted. CuCl and LiO*t*-Bu were purchased from Aldrich Chemical Co., stored under nitrogen, and used as received. Diethoxymethylsilane was purchased from TCI Co., stored under nitrogen, and used as received. *N*,*N*-Dimethylacetamide (DMA) was purchased from Kanto Chemical Co., and stored under argon.

Solvent Screening

Solvent effects were examined with ethyl (*Z*)-3-phenylbut-2-enoate (**1a**) as a substrate using chiral NHC precursor **L1**·HBF_4 (10 mol %), CuCl (10 mol %), KO*t*-Bu (20 mol %), $(TMSO)_2MeSiH$ (1 equiv) as a reductant, and *t*-BuOH (1 equiv) as a protonation reagent at 25 °C for 15 h (Table S1). In general, aprotic polar solvents were appropriate for the conjugate reduction, giving excellent reactivities with good enantioselectivities (Table S1, entries 1–6). Among them, DMA induced the highest enantioselectivity (Table S1, entry 5). On the other hand, the reaction did not proceed at all in nonpolar solvents, toluene, and dichloromethane (Table S1, entries 7 and 8).

Table S1: Optimization of the copper-catalyzed enantioselective conjugate reduction of **1a**.[a]

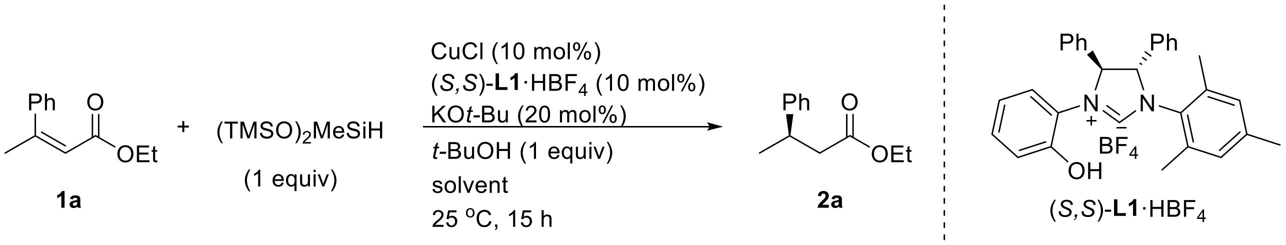

entry	solvent	yield (%)	ee (%)
1	THF	quant	74
2	1,4-dioxane	quant	68
3	MeCN	98%	70
4	DMF	quant	74
5	DMA	quant	76
6	NMP	quant	75
7	toluene	0	–
8	CH_2Cl_2	0	–

[a]Yield was determined by ^1H NMR analysis using 1,1,2,2-tetrachloroethane as an internal standard. Enantiomeric excess (ee) was determined by HPLC analysis with a chiral stationary phase column CHIRALCEL® OD-H.

Preparation of α,β-Unsaturated Esters

General procedure (A) for the (*Z*)-α,β-unsaturated esters

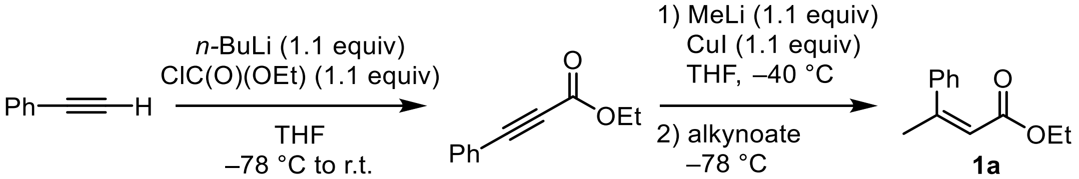

1a

Ethyl (*Z*)-3-phenylbut-2-enoate (1a)

Step 1. To a solution of phenylacetylene (11.8 mL, 100 mmol, 1.0 equiv) in THF (200 mL) was added *n*-BuLi (71.0 mL, 1.55 M in hexanes, 110 mmol, 1.1 equiv) dropwise at −78 °C. After stirring for 1 h at −78 °C, ethyl chloroformate (10.5 mL, 110 mmol, 1.1 equiv) was added dropwise to the resulting mixture and stirred for 1 h at the same temperature. The reaction mixture was gradually warmed to room temperature and stirred overnight. After the addition of saturated NH_4Cl aq, the organic layer was separated, and the aqueous layer was extracted with EtOAc. The combined organic layer was dried over $MgSO_4$, filtered, and the solvent was removed under reduced pressure. The crude product was purified by column chromatography (silica gel, 0–5% EtOAc/hexane) to yield the alkynoate as yellow oil (17.4 g, 99% yield).

Step 2. To a solution of CuI (3.2 g, 15 mmol, 1.1 equiv) in THF was added MeLi (14.9 mL, 1.11 M in Et_2O, 16.5 mmol, 1.1 equiv) dropwise at −40 °C. After stirring for 1 h at −40 °C, the resulting solution was cooled to −78 °C. The alkynoate (2.6 g, 15 mmol, 1.0 equiv) was added dropwise to the solution. After stirring for 6 h at −78 °C, saturated NH_4Cl aq was added to the reaction mixture, and diluted with Et_2O. The mixture was washed with saturated NH_4Cl aq, dried over $MgSO_4$, and filtered. After removal of the solvent under reduced pressure, the crude product was purified by column chromatography (silica gel, 0–5% EtOAc/hexane) to yield **1a** (2.3 g, 82%) as yellow oil. ^{1}H NMR (400 MHz, $CDCl_3$) δ 1.08 (t, *J* = 7.2 Hz, 3H), 2.17 (d, *J* = 1.6 Hz, 3H), 3.99 (q, *J* = 7.2 Hz, 2H), 5.90 (t, *J* = 1.6 Hz, 1H), 7.19-7.21 (m, 2H), 7.28-7.37 (m, 3H). ^{13}C NMR (100.5 Hz, $CDCl_3$) δ 13.9, 27.1, 59.7, 117.7, 126.7, 127.6, 127.8, 140.8, 155.4, 165.9. Spectral data match those reported in the literature.[1]

General procedure (B) for the (*Z*)-α,β-unsaturated Esters

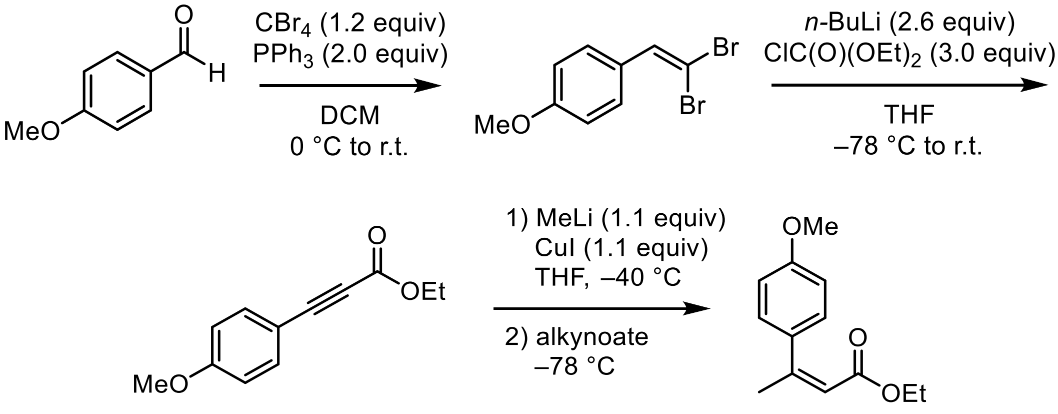

1c

Ethyl (*Z*)-3-(4-methoxyphenyl)but-2-enoate (1c)

Step 1. To a solution of CBr_4 (8.0 g, 24 mmol, 1.2 equiv) in dichloromethane were added PPh_3 (10.7 g, 40 mmol, 2.0 equiv) and 4-methoxybenzaldehyde (2.5 mL, 20 mmol, 1.0 equiv) dropwise at 0 °C. The resulting solution was warmed to room temperature and stirred for 3 h. To the reaction mixture were added hexane and H_2O. The precipitate was filtered through a pad of celite with EtOAc as an eluent. The filtrate was extracted with EtOAc and the combined organic layer was washed with brine. Being dried over $MgSO_4$ and filtered, the solvent was removed under reduced pressure. The crude product was purified by column chromatography (silica gel, 0–5-8% EtOAc/hexane) to yield 1,1-dibromoalkene as a pale yellow solid (4.0 g, 68% yield).

Step 2. To a solution of 1,1-dibromoalkene in THF was added *n*-BuLi (23.2 mL, 1.55 M in hexanes, 35.9 mmol, 2.6 equiv) dropwise at –78 °C. After stirring for 1 h at –78 °C, the reaction mixture was gradually warmed to room temperature for 1 h. Ethyl chloroformate (3.9 mL, 41.4 mmol, 3.0 equiv) was added to the reaction solution at –78 °C, then the mixture was warmed to room temperature and stirred for overnight. After the addition of saturated NH_4Cl aq, the mixture was separated, and the aqueous layer was extracted with EtOAc. Combined organic layer was washed with brine, dried over $MgSO_4$, and filtered. After removal of the solvent under the reduced pressure, the crude product was purified by column chromatography (silica gel, 0–7% EtOAc/hexane) to yield the alkynoate as yellow oil (2.4 g, 84% yield).

Step 3. Following the same procedure as the Step 2 in the general procedure A, **1c** was obtained as pale yellow oil (1.6 g, 63% yield). ^{1}H NMR (400 MHz, $CDCl_3$) δ 1.14 (t, J = 7.2 Hz, 3H), 2.16 (d, J = 1.2 Hz, 3H), 3.81 (s, 3H), 4.03 (q, J = 7.2 Hz, 2H), 5.87 (t, J = 1.2 Hz, 1H), 6.88 (td, J = 2.4 Hz, 8.8 Hz, 2H), 7.19 (td, J = 2.4 Hz, 8.8 Hz, 2H). ^{13}C NMR (100.5 Hz, $CDCl_3$) δ 14.1, 27.1, 55.2, 59.7, 113.2, 117.0, 128.5, 132.6, 154.9, 159.3, 166.1.

Spectral data match those reported in the literature.[2]

Procedure for the (*Z*)-β,β-diaryl-substituted-α,β-unsaturated ester

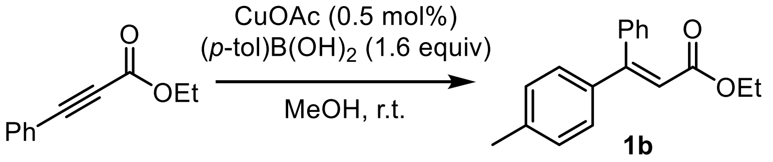

Ethyl (*E*)-3-phenyl-3-(*p*-tolyl)acrylate (1b)

To a solution of CuOAc (1.2 mg, 2.0 mmol, 0.5 mol %) and the boronic acid (435 mg, 3.2 mmol, 1.6 equiv) in MeOH was added the alkynoate (348 mg, 2.0 mmol, 1.0 equiv) at room temperature. After stirring overnight, the resulting mixture was filtered through a short plug of silica gel with diethyl ether as an eluent. The solvent was removed under reduced pressure and the crude product was purified by column chromatography (silica gel, 0–5% EtOAc/hexane) to yield **1b** as pale yellow oil (236 mg, 44% yield). ^{1}H NMR (400 MHz, $CDCl_3$) δ 1.10 (t, J = 7.2 Hz, 3H), 2.36 (s, 1H), 4.04 (q, J = 7.2 Hz, 2H), 6.35 (s, 1H), 7.12 (d, J = 8.0 Hz, 2H), 7.18-7.22 (m, 4H) 7.35-7.39 (m, 3H). ^{13}C NMR (100.5 Hz, $CDCl_3$) δ 14.0, 21.2, 60.0, 116.5, 127.8, 128.0, 128.2, 129.1, 137.9, 139.1, 139.7, 156.5, 166.2.

Spectral data match those reported in the literature.[3]

Procedure for the (*Z*)-α,β-unsaturated Amide

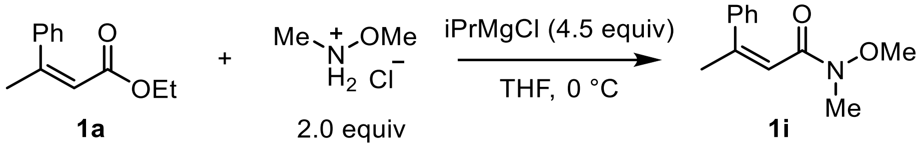

(*Z*)-*N*-Methoxy-*N*-methyl-3-phenylbut-2-enamide (1i)

To a solution of **1a** (285 mg, 1.5 mmol, 1.0 equiv) and *N*,*O*-dimethylhydroxylammonium chloride (302 mg, 3.0 mmol, 2.0 equiv) in THF was added iPrMgCl (5.3 mL, 1.28 M in THF, 6.75 mmol, 4.5 equiv) dropwise at 0 °C. After stirring for 3 h at 0 °C, the reaction mixture was quenched with saturated NH_4Cl aq and extracted with EtOAc. The combined organic layer was washed with brine, dried over $MgSO_4$ and filtered. The solvent was removed under reduced pressure, then the crude product was purified by column chromatography (silica gel, 10–20% EtOAc/hexane) to yield **1i** as pale yellow oil (280 mg, 91% yield). ^{1}H NMR (400 MHz, $CDCl_3$) δ 2.18 (s, 3H), 3.08 (brs, 3H), 3.64 (brs, 3H), 6.28 (brs, 1H), 7.24-7.34 (m, 5H). ^{13}C NMR (100.5 Hz, $CDCl_3$) δ 26.5, 31.8, 61.2, 116.7, 126.6, 127.4, 127.7, 140.7, 150.7, 167.1. IR (ATR) 697, 764, 828, 982, 1025, 1113, 1176, 1334, 1381, 1442, 1654, 2937, 2972. HRMS–EI (m/z): [M]+ calcd for $C_{12}H_{15}NO_2$, 205.1103; found, 205.1098.

Procedure for the (*E*)-α,β-unsaturated ester

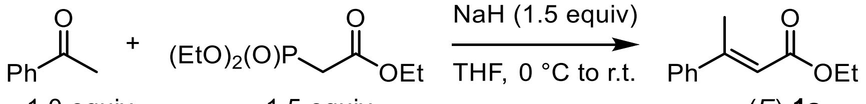

Ethyl (*E*)-3-phenylnut-2-enoate ((*E*)-1a)

To a solution of NaH (981 mg, 22.5 mmol, 1.5 equiv) in THF (70 mL) was added triethyl phosphonoacetate (4.6 mL, 22.5 mmol, 1.5 equiv) dropwise at 0 °C. After completion of the generation of H_2 gas, acetophenone (1.78 mL, 15 mmol, 1.0 equiv) was added to the reaction mixture. After being stirred at room temperature for 6 h, the resulting mixture was quenched with saturated NH_4Cl aq. The mixture was extracted with EtOAc. The combined organic layer was washed with brine, dried over $MgSO_4$ and filtered. After removal of the solvent under vacuum, the crude product was purified by column chromatography (silica gel, 0–1% EtOAc/hexane) to yield (*E*)-**1a** as pale yellow oil (1.73 g, 60% yield). ^{1}H NMR (400 MHz, $CDCl_3$) δ 1.31 (t, *J* = 7.2 Hz, 3H), 2.58 (d, *J* = 1.6 Hz, 3H), 4.21 (q, *J* = 7.2 Hz, 2H), 6.14 (t, *J* = 1.6 Hz, 1H), 7.34-7.39 (m, 3H), 7.45-7.49 (m, 2H). ^{13}C NMR (100.5 Hz, $CDCl_3$) δ 14.3, 17.9, 59.8, 117.1, 126.2, 128.4, 128.9, 142.2, 155.5, 166.8. Spectral data match those reported in the literature.[1]

Characterization Data for α,β-Unsaturated Esters

Ethyl (*Z*)-3-(4-fluorophenyl)but-2-enoate (1d)

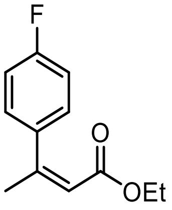

Synthesized from 4-fluorobenzaldehyde with the general procedure B. 1.7 g, 28% yield in 3 steps (20 mmol). Yellow oil. ^{1}H NMR (400 MHz, $CDCl_3$) δ 1.11 (t, *J* = 7.2 Hz, 3H), 2.16 (d, *J* = 1.2 Hz, 3H), 4.01 (q, *J* = 7.2 Hz, 2H), 5.91 (d, *J* = 1.2 Hz, 1H), 7.03 (tt, *J* = 1.5 Hz, 8.8 Hz, 2H), 7.17-7.20 (m, 2H). ^{13}C NMR (100.5 Hz, $CDCl_3$) δ 14.0, 27.2, 59.8, 114.8 (d, *J* = 21.0 Hz), 118.0, 128.7 (d, *J* =8.6 Hz), 136.5 (d, *J* = 2.8 Hz), 154.3, 162.2 (d, *J* = 246.0 Hz), 165.8. IR (ATR) 834, 1045, 1153, 1167, 1225, 1278, 1376, 1443, 1508, 1603, 1639, 1719, 2981. HRMS–EI (m/z): [M]+ calcd for $C_{12}H_{13}O_2F$, 208.0900; found, 208.0897.

Ethyl (*Z*)-5-(4-methoxy-4-oxobut-2-en-2-yl)thiophene-2-carboxylate (1e)

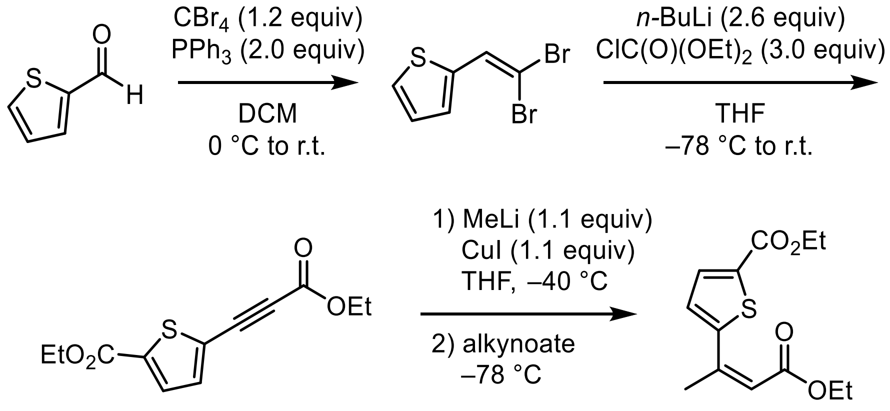

1e

Synthesized from thiophene-2-carbaldehyde with the general procedure B. 413 mg, 8% yield in 3 steps (20 mmol). Orange solid. ^{1}H NMR (400 MHz, $CDCl_3$) δ 1.23 (dt, *J* = 1.2 Hz, 7.2 Hz, 3H), 1.37 (dt, *J* = 1.2 Hz, 7.2 Hz, 3H), 2.27 (d, *J* = 1.2 Hz, 3H), 4.14 (dq, *J* = 1.2 Hz, 7.2 Hz, 2H), 4.34 (dq, *J* = 1.2 Hz, 7.2 Hz, 2H), 5.96 (d, *J* = 1.2 Hz, 1H), 7.32 (d, *J* = 3.6 Hz, 1H), 7.68-7.70 (m, 1H). ^{13}C NMR (100.5 Hz, $CDCl_3$) δ 14.0, 14.3, 27.2, 60.2, 61.1, 119.1, 128.7, 132.6, 134.3, 143.7, 146.8, 162.1, 165.4. IR (ATR) 752, 822, 853, 1097, 1173, 1212, 1252, 1366, 1441, 1520, 1609, 1702, 2979. HRMS–EI (m/z): [M]+ calcd for $C_{13}H_{16}O_4S$, 268.0769; found, 268.0770.

Ethyl (*Z*)-3-methyl-5-phenylpent-2-enoate (1f)

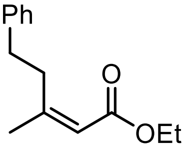

Synthesized from 4-phenyl-1-butyne with the general procedure A. 2.5 g, 76% yield in 2 steps (15 mmol). Colorless oil. ^{1}H NMR (400 MHz, $CDCl_3$) δ 1.27 (t, *J* = 7.2 Hz, 3H), 1.88 (d, *J* = 1.2 Hz, 3H), 2.78 (m, 2H), 2.91 (m, 2H), 4.14 (q, *J* = 7.2 Hz, 2H), 5.69 (d, *J* = 1.2 Hz, 1H), 7.16-7.30 (m, 5H). ^{13}C NMR (100.5 Hz, $CDCl_3$) δ 14.3, 25.5, 34.5, 35.5, 59.5, 116.6, 125.9, 127.6, 128.3, 128.4, 141.6, 159.5, 166.2.

Spectral data match those reported in the literature.[4]

Benzyl (*Z*)-3-phenylbut-2-enoate (1g)

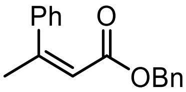

Synthesized with the general procedure A using benzyl chloroformate. 570 mg, 44% yield in 2 steps (5 mmol). Colorless oil. ^{1}H NMR (400 MHz, $CDCl_3$) δ 2.17 (d, *J* = 1.2 Hz, 3H), 4.99 (s, 2H), 5.96 (t, *J* = 1.2 Hz, 1H), 7.12-7.14 (m, 2H), 7.18-7.22 (m, 2H), 7.25-7.35 (m, 6H). ^{13}C NMR (100.5 Hz,

$CDCl_3$) δ 27.2, 65.6, 117.3, 126.8, 127.7, 127.8, 127.9, 128.0, 128.3, 128.5, 135.9, 140.7, 156.0, 165.6. IR (ATR) 695, 738, 1026, 1150, 1226, 1264, 1637, 1723, 3032. HRMS–EI (m/z): [M]+ calcd for $C_{17}H_{16}O_2$, 252.1150; found, 252.1144.

Isopropyl (*Z*)-3-phenylbut-2-enoate (1h)

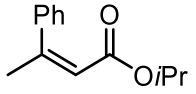

Synthesized with the general procedure A using methyl chloroformate. 776 mg, 33% yield in 2 steps (5 mmol). Pale yellow oil. ^{1}H NMR (400 MHz, $CDCl_3$) δ 1.05 (d, *J* = 6.0 Hz, 6H), 2.17 (d, *J* = 1.2 Hz, 3H), 4.87 (sep, *J* = 6.0 Hz, 1H), 5.88 (q, *J* = 1.2 Hz, 1H), 7.18-7.21 (m, 2H), 7.28-7.36 (m, 3H). ^{13}C NMR (100.5 Hz, $CDCl_3$) δ 21.5, 27.0, 67.0, 118.3, 126.8, 127.5, 127.8, 140.9, 154.6, 165.4. IR (ATR) 697, 766, 1024, 1107, 1165, 1230, 1277, 1374, 1442, 1637, 1702, 2979. HRMS–EI (m/z): [M]+ calcd for $C_{13}H_{16}O_2$, 204.1150; found, 204.1148.

Procedure for the Copper-catalyzed Enantioselective Conjugate Reduction

In a glove box, CuCl (1.5 mg, 0.015 mmol), **L4**·HBF_4 (9.8 mg, 0.015 mmol), and LiO*t*-Bu (2.4 mg, 0.03 mmol) were placed in a vial containing a magnetic stirring bar. The vial was sealed with a teflon-coated silicon rubber septum. *N*,*N*-Dimethylacetamide (0.90 mL) was added to the vial, and then the mixture was stirred at room temperature for 5 min. Next, *t*-AmOH (16.3 μL, 0.15 mmol) was added and stirred at the same temperature, and the vial was taken out of the glove box. To the reaction mixture was added α,β-unsaturated ester (0.15 mmol). After stirring for 5 min at room temperature, diethoxymethylsilane (100.9 μL, 0.60 mmol) was added to the mixture. After stirring for 15 h at 25 °C, the reaction mixture was diluted with diethyl ether (1.0 mL) and quenched with H_2O (0.6 mL). The organic layer was separated, and the aqueous layer was extracted three times. The combined organic layer was filtered through a short plug of silica gel with diethyl ether as an eluent. After the solvent was removed under reduced pressure, the yields of products were determined by ^{1}H NMR with 1,1,2,2-tetrachloroethane as an internal standard. The pure product was obtained after the purification by GPC (eluent: $CHCl_3$).

Characterization Data for Reduction Products

Ethyl (*R*)-3-phenylbutanoate (2a)

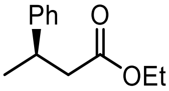

Colorless oil. 15.1 mg, 52%, 90% ee. ^{1}H NMR (400 MHz, $CDCl_3$) δ 1.17 (t, J = 7.2 Hz, 3H), 1.29 (d, J = 7.2 Hz, 3H), 2.50-2.63 (m, 2H), 3.27 (sext, J = 7.2 Hz, 1H), 4.06 (q, J = 7.2 Hz, 2H), 7.16-7.22 (m, 3H), 7.28 (t, J = 7.2 Hz, 2H). ^{13}C NMR (100.5 Hz, $CDCl_3$) δ 14.1, 21.7, 36.4, 42.9, 60.2, 126.3, 126.7, 128.4, 145.6, 172.3.

Spectral data match those reported in the literature.[5]

$[\alpha]_{27.3}{}^{D}$ –23.2 (*c* 1.25, $CHCl_3$).

The ee value was determined by chiral HPLC analysis (CHIRALCEL® OD-H column, 4.6 mm 250 mm Daicel Chemical Industries, hexane/2-propanol = 98:2, 0.5 mL/min, 40 °C, 220 nm UV detector, retention time = 9.1 min for (*R*)-isomer and 13.2 min for (*S*)-isomer). The absolute configuration of **2a** was assigned by the comparison of the optical rotation with the same compound prepared by reported method.[6]

Ethyl (*R*)-3-phenyl-3-(*p*-tolyl)propanoate (2b)

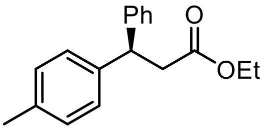

Colorless oil. 23.9 mg, 60%, 75% ee. ^{1}H NMR (400 MHz, $CDCl_3$) δ 1.11 (t, J = 7.2 Hz, 3H), 2.28 (s, 3H), 3.03 (d, J = 8.0 Hz, 2H), 4.02 (q, J = 7.2 Hz, 2H), 4.51 (t, J = 8.0 Hz, 1H), 7.06-7.18 (m, 5H), 7.21-7.28 (m, 4H). ^{13}C NMR (100.5 Hz, $CDCl_3$) δ 14.0, 21.0, 40.8, 46.6, 60.4, 126.4, 127.5, 127.6, 128.5, 129.2, 136.0, 140.5, 143.7, 171.9.

Spectral data match those reported in the literature.[3]

$[\alpha]_{27.3}{}^{D}$ –2.57 (*c* 1.06, $CHCl_3$).

The ee value was determined by chiral HPLC analysis (CHIRALCEL® OD-3 column, 4.6 mm 250 mm Daicel Chemical Industries, hexane/2-propanol = 99:1, 1.0 mL/min, 220 nm UV detector, retention time = 7.1 min for (*R*)-isomer and 9.8 min for (*S*)-isomer). The absolute configuration of **2b** was assigned by the comparison of HPLC analysis of the reported condition.[3]

Ethyl (*R*)-3-(4-methoxyphenyl)butanoate (2c)

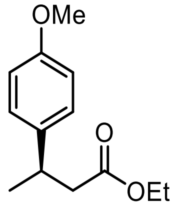

Pale yellow oil. 14.4 mg, 44%, 84% ee. ^{1}H NMR (400 MHz, $CDCl_3$) δ 1.18 (t, *J* = 7.2 Hz, 3H), 1.27 (d, *J* = 7.2 Hz, 3H), 2.47-2.60 (m, 2H), 3.23 (sext, *J* = 7.2 Hz, 1H), 3.78 (s, 3H), 4.07 (q, *J* = 7.2 Hz, 2H), 6.84 (td, *J* = 2.4 Hz, 8.8 Hz, 2H), 7.14 (td, *J* = 2.4 Hz, 8.8 Hz, 2H). ^{13}C NMR (100.5 Hz, $CDCl_3$) δ 14.1, 21.9, 35.7, 43.2, 55.2, 60.2, 113.7, 127.6, 137.8, 158.0, 172.4.

Spectral data match those reported in the literature.[5]

$[\alpha]_{27.3}^{D}$ –23.1 (*c* 1.46, $CHCl_3$).

The ee value was determined by chiral HPLC analysis (CHIRALCEL® OD-H column, 4.6 mm 250 mm Daicel Chemical Industries, hexane/2-propanol = 99:1, 0.5 mL/min, 40 °C, 220 nm UV detector, retention time = 14.2 min for (*R*)-isomer and 16.9 min for (*S*)-isomer).

Ethyl (*R*)-3-(4-fluorophenyl)butanoate (2d)

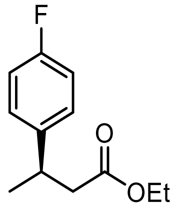

Pale yellow oil. 23.6 mg, 74%, 76% ee. ^{1}H NMR (400 MHz, $CDCl_3$) δ 1.17 (t, *J* = 7.2 Hz, 3H), 1.28 (d, *J* = 7.2 Hz, 3H), 2.49-2.60 (m, 2H), 3.27 (sext, *J* = 7.2 Hz, 1H), 4.06 (q, *J* = 7.2 Hz, 2H), 6.98 (tt, *J* = 2.4 Hz, 8.8 Hz, 2H), 7.15-7.20 (m, 2H). ^{13}C NMR (100.5 Hz, $CDCl_3$) δ 14.1, 22.0, 35.8, 43.1, 60.3, 115.1 (d, *J* = 20.9 Hz), 128.1 (d, *J* = 8.6 Hz), 141.3 (d, *J* = 2.9 Hz), 161.4 (d, *J* = 243.1 Hz), 172.2.

Spectral data match those reported in the literature.[5]

$[\alpha]_{27.3}^{D}$ –19.7 (*c* 1.62, $CHCl_3$).

The ee value was determined by chiral HPLC analysis (CHIRALCEL® IG-3 column, 4.6 mm 250 mm Daicel Chemical Industries, hexane/2-propanol = 99.5:0.5, 0.5 mL/min, 40 °C, 220 nm UV detector, retention time = 22.2 min for (*S*)-isomer and 22.9 min for (*R*)-isomer).

Ethyl (*R*)-5-(4-ethoxy-oxobutan-2-yl)thiophene-2-carboxylate (2e)

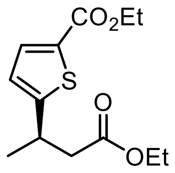

Colorless oil. 30.6 mg, 75%, 84% ee. ^{1}H NMR (400 MHz, $CDCl_3$) δ 1.23 (t, *J* = 7.2 Hz, 3H), 1.36 (t, *J* = 7.2 Hz, 3H), 1.39 (d, *J* = 7.2 Hz, 3H), 2.55-2.70 (m, 2H), 3.59 (sext, *J* = 7.2 Hz, 1H), 4.12 (q, *J* = 7.2 Hz, 2H), 4.32 (q, *J* =7.2 Hz, 2H), 6.85 (d, *J* = 4.0 Hz, 1H), 7.63 (d, *J* = 4.0 Hz, 1H). ^{13}C NMR (100.5 Hz, $CDCl_3$) δ 14.1, 14.3, 22.4, 32.4, 43.4, 60.6, 61.0, 124.0, 131.2, 133.3, 157.3, 162.2, 171.4. IR (ATR)749, 818, 1029, 1088, 1173, 1255, 1280, 1369, 1465, 1705, 1733, 2980. HRMS–EI (m/z): [M]+ calcd for $C_{13}H_{18}O_4S$, 270.0926; found, 270.0927.

$[\alpha]_{27.5}^{D}$ –29.1 (*c* 1.46, $CHCl_3$).

The ee value was determined by chiral HPLC analysis (CHIRALCEL® OJ-3 column, 2.1 mm 250 mm Daicel Chemical Industries, and OJ-H column, 4.6 mm 250 mm Daicel Chemical Industries, hexane/2-propanol = 99:1, 0.5 mL/min, 40 °C, 250 nm UV detector, retention time = 45.7 min for (*S*)-isomer and 49.2 min for (*R*)-isomer).

Ethyl (*R*)-3-methyl-5-phenylpentanoate (2f)

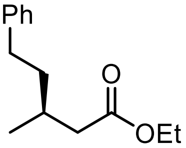

Colorless oil. 14.8 mg, 45%, 85% ee. ^{1}H NMR (400 MHz, $CDCl_3$) δ 1.01 (d, *J* =7.2 Hz, 3H), 1.25 (t, *J* = 7.2 Hz, 3H), 1.46-1.58 (m, 1H), 1.62-1.71 (m, 1H), 2.02 (oct, *J* = 7.2 Hz, 1H), 2.16 (dd, *J* = 8.0 Hz, 14.4 Hz, 1H), 2.34 (dd, *J* = 6.0 Hz, 14.4 Hz, 1H), 2.54-2.69 (m, 2H), 4.12 (q, *J* = 7.2 Hz, 2H), 7.15-7.19 (m, 3H), 7.25-7.29 (m, 2H). ^{13}C NMR (100.5 Hz, $CDCl_3$) δ 14.3, 19.6, 30.1, 33.3, 38.5, 41.8, 60.2, 125.7, 128.2, 128.3, 142.4, 173.1.

Spectral data match those reported in the literature.[7] The ee value was determined by chiral HPLC analysis of 3-methyl-5-phenylpentan-1-ol obtained by the reduction of **2e** using DIBAL-H (Scheme S1) (CHIRALCEL® OD-H column, 4.6 mm 250 mm Daicel Chemical Industries, hexane/2-propanol = 97:3, 0.5 mL/min, 40 °C, 220 nm UV detector, retention time = 24.0 min for *S*-isomer and 26.4 min for (*R*)-isomer).

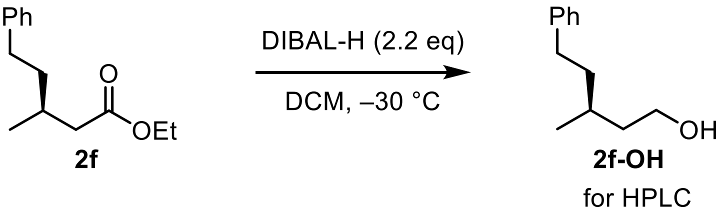

Scheme S1. Transformation of **2f**. **2f-OH**: $[\alpha]_{27.3}^{D}$ –11.0 (*c* 0.40, $CHCl_3$).

Benzyl (*R*)-3-phenylbutanoate (2g)

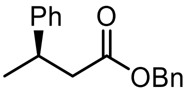

Colorless oil. 20.9 mg, 55%, 70% ee. ^{1}H NMR (400 MHz, $CDCl_3$) δ 1.29 (d, *J* = 7.2 Hz, 3H), 2.57-2.71 (m, 2H), 3.29 (sext, *J* = 7.2 Hz, 1H), 5.05 (s, 2H), 7.18-7.24 (m, 5H), 7.26-7.35 (m, 5H). ^{13}C NMR (100.5 Hz, $CDCl_3$) δ 21.9, 36.5, 42.9, 66.1, 126.4, 126.7, 128.1, 128.5, 135.8, 145.5, 172.2.

Spectral data match those reported in the literature.[8]

$[\alpha]_{27.3}^{D}$ –17.4 (*c* 1.35, $CHCl_3$).

The ee value was determined by chiral HPLC analysis (CHIRALCEL® OD-H column, 4.6 mm 250 mm Daicel Chemical Industries, hexane/2-propanol = 98:2, 0.5 mL/min, 40 °C, 220 nm UV detector, retention time = 12.5 min for (*R*)-isomer and 19.6 min for (*S*)-isomer).

Isopropyl (*R*)-3-phenylbutanoate (2h)

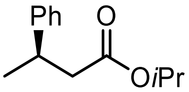

Colorless oil. 24.4 mg, 79%, 83% ee. ^{1}H NMR (400 MHz, $CDCl_3$) δ 1.11 (d, *J* = 6.4 Hz, 3H), 1.16 (d, *J* = 6.4 Hz, 3H), 1.29 (d, *J* = 7.2 Hz, 3H), 2.48-2.61 (m, 2H), 3.26 (sext, *J* = 7.2 Hz, 1H), 4.94 (sep, *J* = 6.4 Hz, 1H), 7.17-7.23 (m, 3H), 7.27-7.31 (m, 2H). ^{13}C NMR (100.5 Hz, $CDCl_3$) δ 21.6, 21.7, 21.8, 36.6, 43.3, 67.5, 126.3, 126.8, 128.4, 145.7, 171.9.

Spectral data match those reported in the literature.[9]

$[\alpha]_{27.2}^{D}$ –21.4 (*c* 1.17, $CHCl_3$).

The ee value was determined by chiral HPLC analysis (CHIRALCEL® OD-H column, 4.6 mm 250 mm Daicel Chemical Industries, hexane/2-propanol = 98:2, 0.5 mL/min, 40 °C, 220 nm UV detector, retention time = 8.1 min for (*R*)-isomer and 8.9 min for (*S*)-isomer).

(*R*)-*N*-Methoxy-*N*-methyl-3-phenylbutanamide (2i)

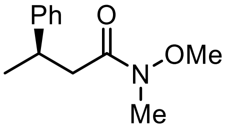

Pale yellow oil. 19.0 mg, 59%, 79% ee. ^{1}H NMR (400 MHz, $CDCl_3$) δ 1.32 (d, J = 7.2 Hz, 3H), 2.62-2.77 (m, 2H), 3.14 (s, 3H), 3.37 (sext, J = 7.2 Hz, 1H), 3.58 (s, 3H), 7.17-7.22 (m, 1H), 7.25-7.32 (m, 4H). ^{13}C NMR (100.5 Hz, $CDCl_3$) δ 21.7, 32.0, 35.8, 40.3, 61.2, 126.2, 126.9, 128.4, 146.5, 173.1.

Spectral data match those reported in the literature.[10]

$[\alpha]_{27.3}^{D}$ 3.00 (*c* 1.15, $CHCl_3$).

The ee value was determined by chiral HPLC analysis (CHIRALCEL® OD-H column, 4.6 mm 250 mm Daicel Chemical Industries, hexane/2-propanol = 98:2, 0.5 mL/min, 40 °C, 220 nm UV detector, retention time = 16.7 min for (*S*)-isomer and 17.7 min for (*R*)-isomer).

References

1) Fronza, G.; Fuganti, C.; Serra, S. *Eur. J. Org. Chem.* **2009**, *35*, 6160.

2) Scheiper, B.; Bonnekessel, M.; Krause, H.; Fürstner, A. *J. Org. Chem.* **2004**, *69*, 3943.

3) Itoh, K.; Tsuruta, A.; Ito, J.; Yamamoto, Y.; Nishiyama, H. *J. Org. Chem.* **2012**, *77*, 10914.

4) Katsukiyo, M.; Masaru, E.; Kazunori, O.; Junji, I.; Akira, H. *Chem. Lett.* **2009**, *38*, 832.

5) Guo, S.; Zhou, J. *Org. Lett.* **2016**, *18*, 5344.

6) Appella, D. H.; Moritani, Y.; Shintani, R.; Ferreira, E. M.; Buchwald, S. L. *J. Am. Chem. Soc.* **1999**, *121*, 9473.

7) Lipshutz, B. H.; Servesko, J. M.; Taft, B. R. *J. Am. Chem. Soc.* **2004**, *126*, 8352.

8) Yuan, P.; Chen, J.; Zhao, J.; Huang, Y. *Angew. Chem. Int. Ed.* **2018**, *57*, 8503.

9) Oi, S.; Taira, A.; Honma, Y.; Sato, T.; Inoue, Y. *Tetrahedron Asymmetry* **2006**, *17*, 598.

10) Shintani, R.; Kimura, T.; Hayashi, T. *Chem. Commun.* **2005**, 3213.

HPLC Charts

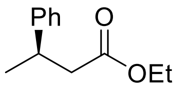

2a

racemic

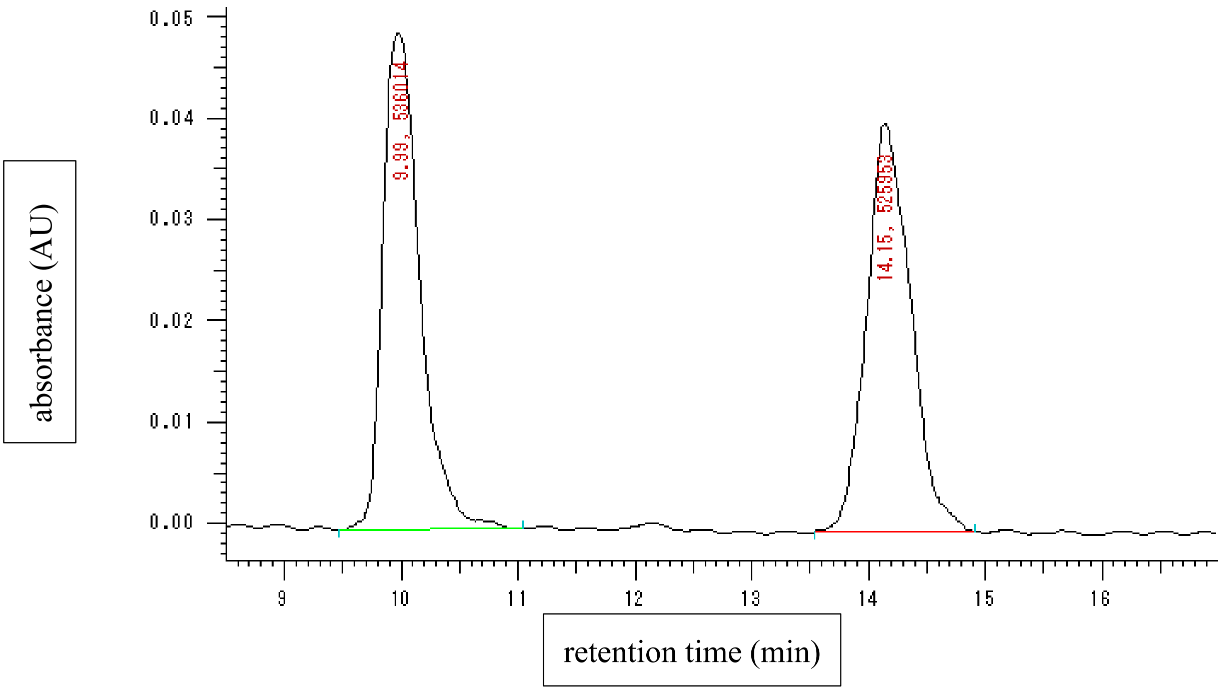

chiral

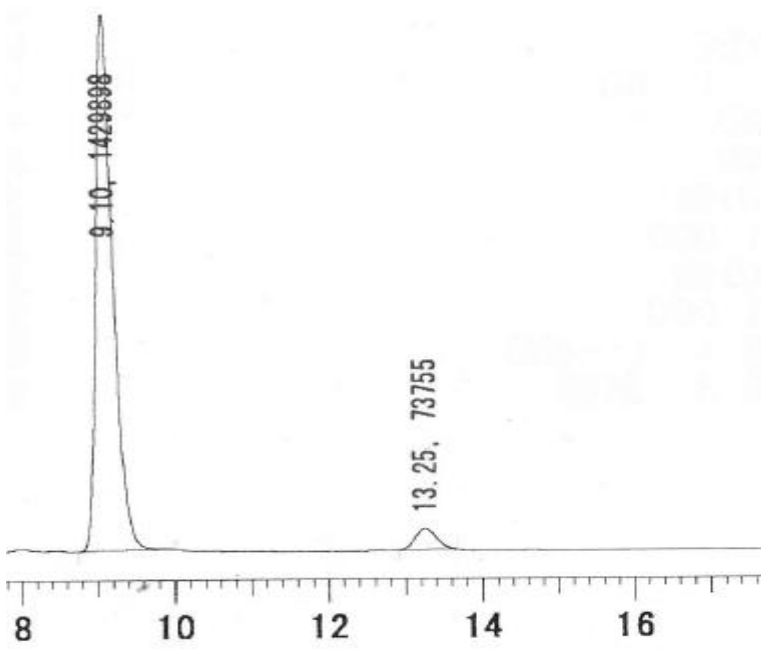

(*S*)-**2a**

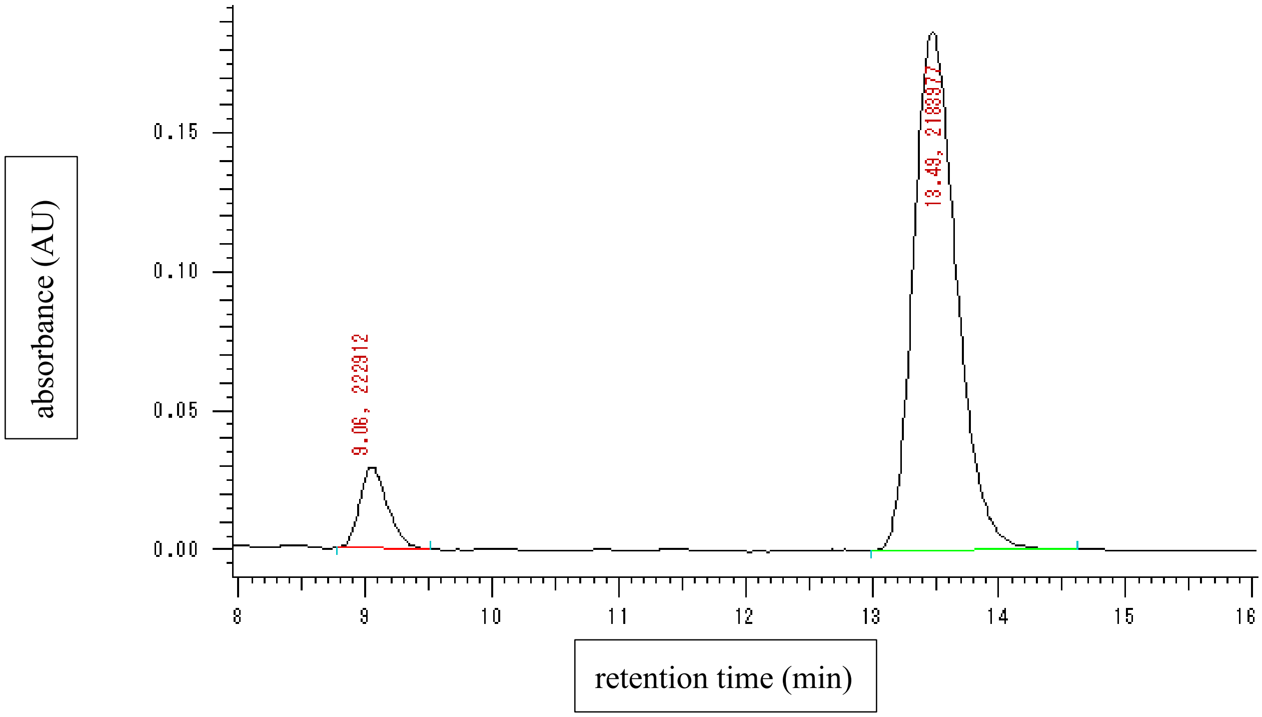

racemic				chiral				(*S*)-**2a**			
no.	RT (min)	area	area (%)	no.	RT (min)	area	area (%)	no.	RT (min)	area	area (%)
1	9.99	536014	50.474	1	9.10	1429898	95.095	1	9.06	222912	9.336
2	14.15	525953	49.526	2	13.25	73755	4.905	2	13.49	2164843	90.664

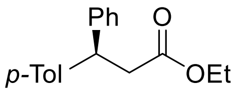

2b

racemic

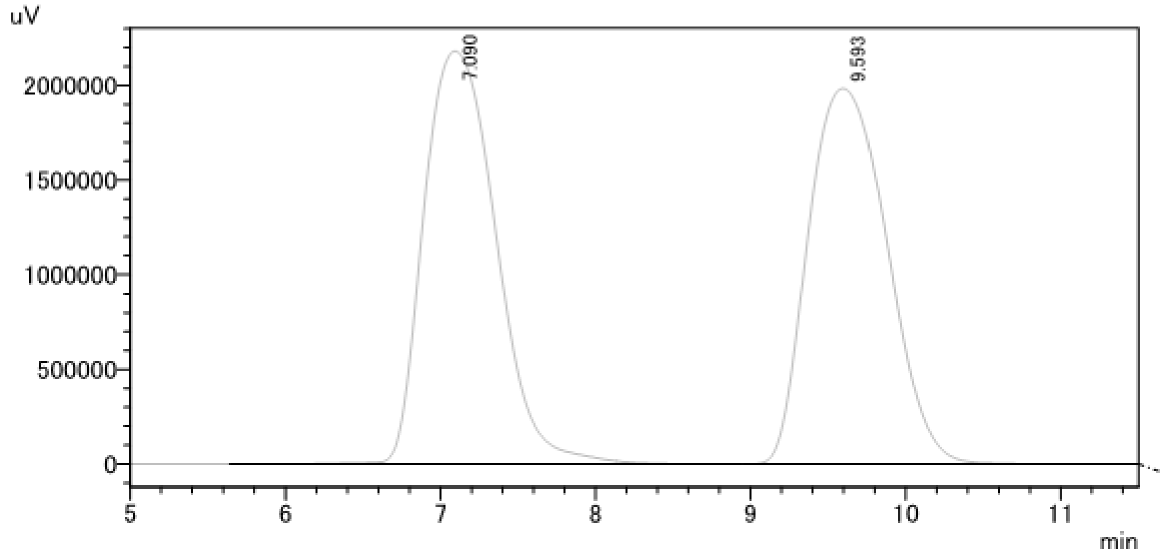

chiral

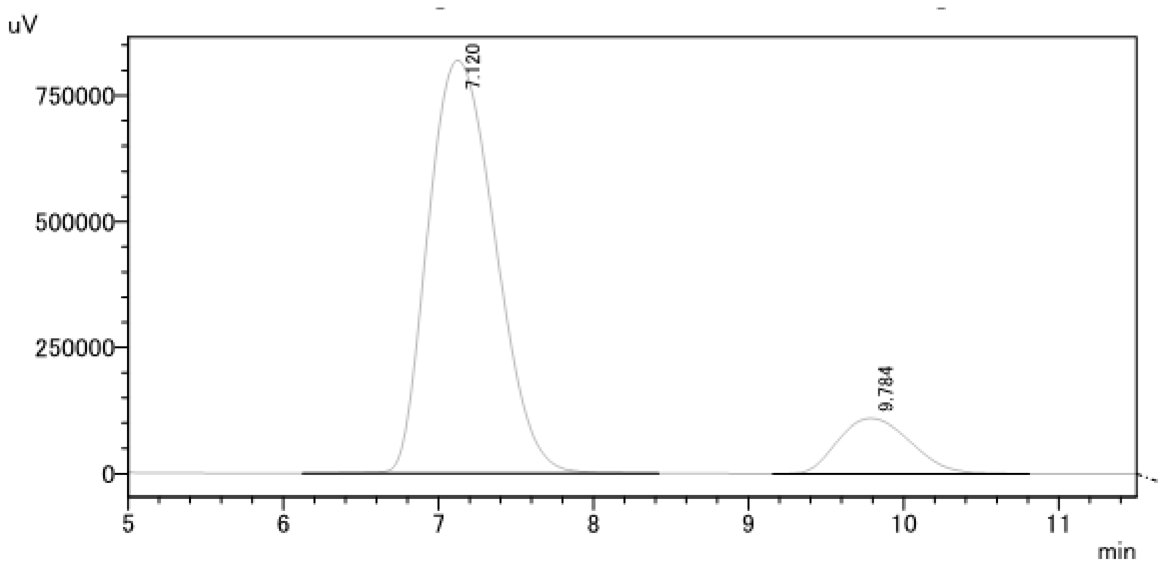

racemic				chiral			
no.	RT (min)	area	area (%)	no.	RT (min)	area	area (%)
1	7.090	70690499	50.267	1	7.120	24674408	87.454
2	9.593	69939532	49.733	2	9.784	3539639	12.546

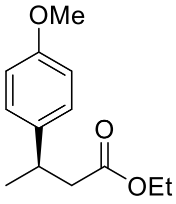

2c

racemic

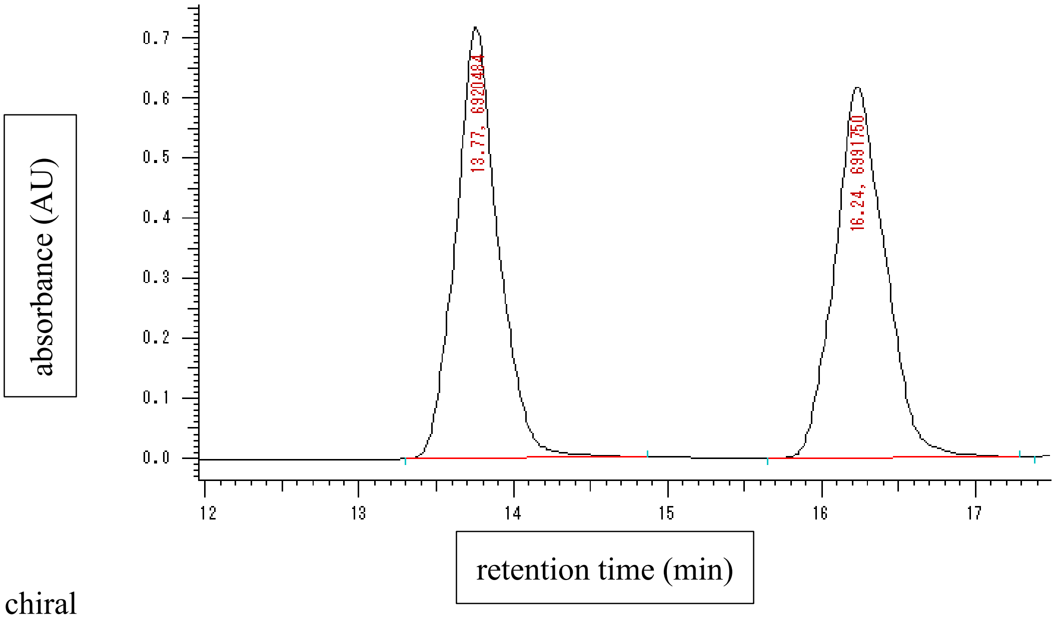

chiral

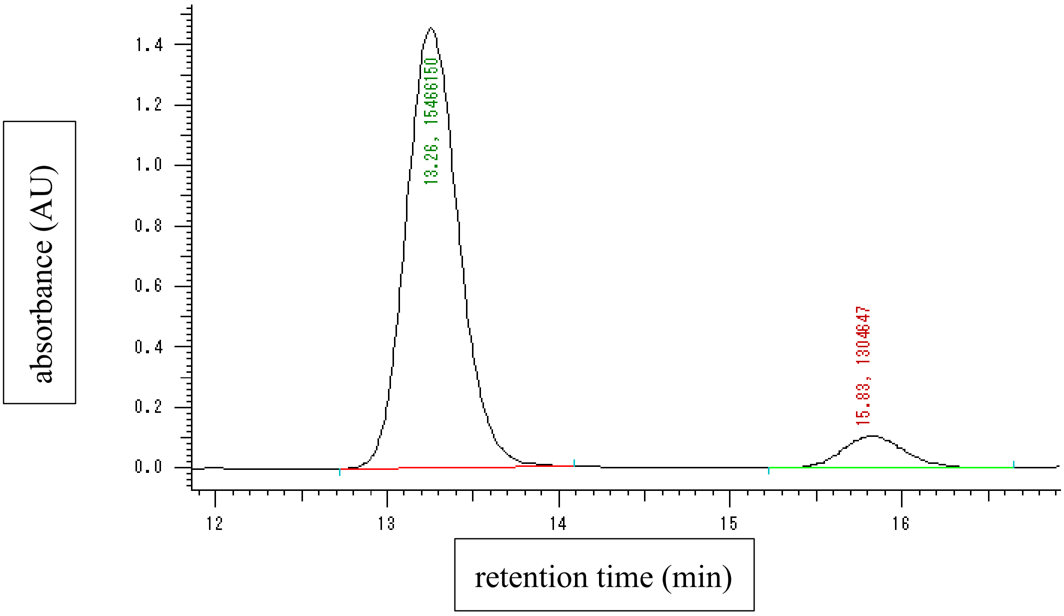

racemic				chiral			
no.	RT (min)	area	area (%)	no.	RT (min)	area	area (%)
1	13.77	6920484	49.744	1	13.26	15466150	92.221
2	16.24	699.1750	50.256	2	15.83	1304647	7.779

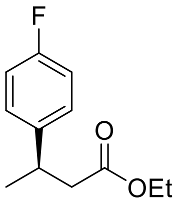

2d

racemic

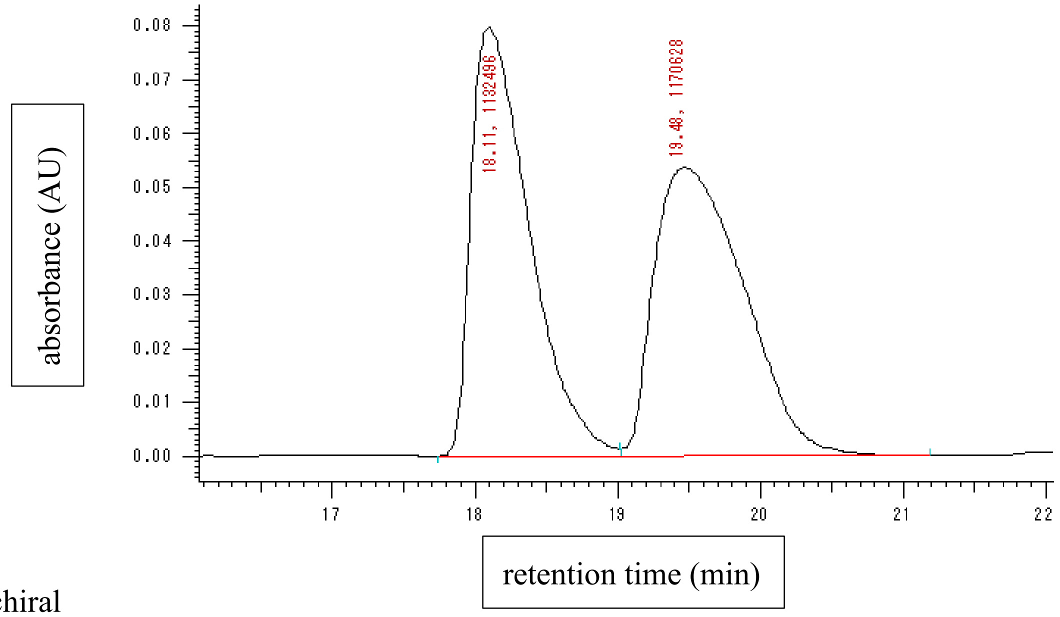

chiral

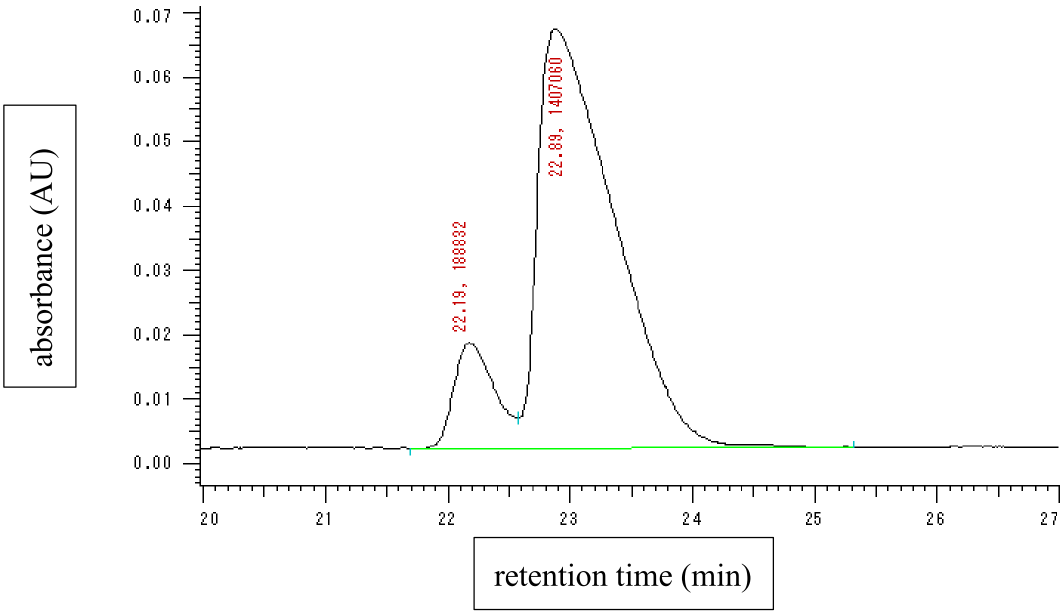

racemic / chiral

no.	RT (min)	area	area (%)	no.	RT (min)	area	area (%)
1	18.11	1132496	49.172	1	22.19	188832	11.832
2	19.48	1170628	50.828	2	22.89	1407060	88.168

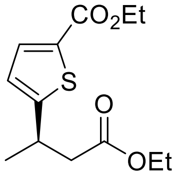

2e

racemic

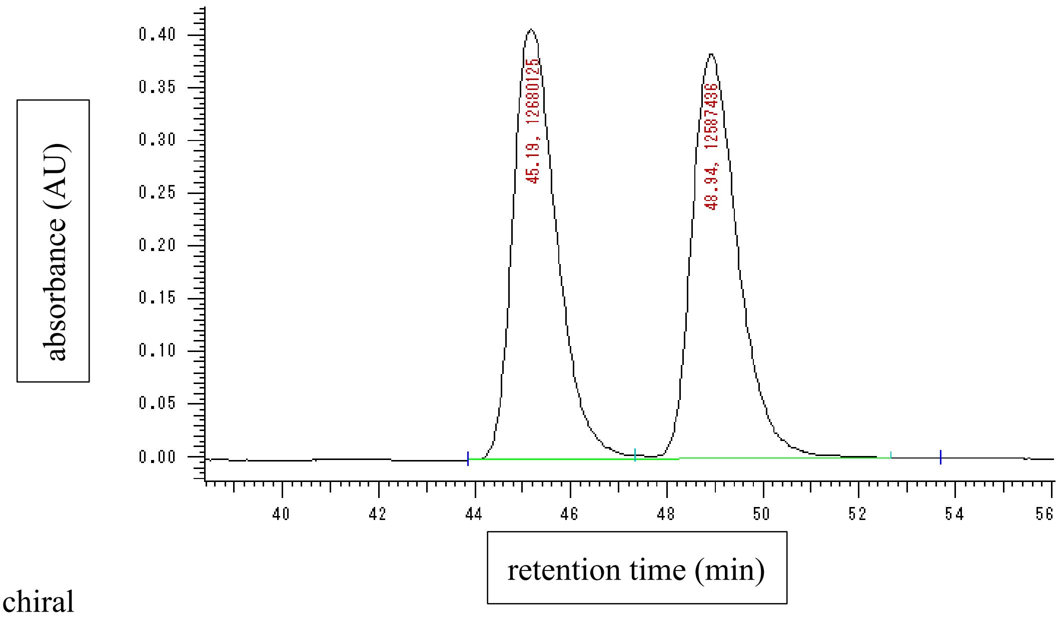

chiral

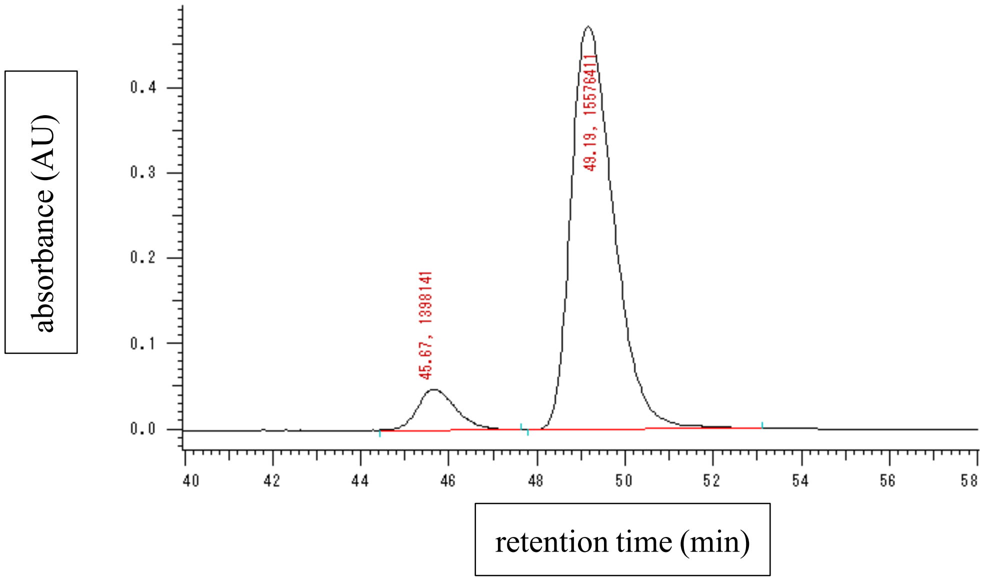

racemic				chiral			
no.	RT (min)	area	area (%)	no.	RT (min)	area	area (%)
1	45.19	12680125	50.183	1	45.67	1398141	8.237
2	48.94	12587436	49.817	2	49.19	15576411	91.763

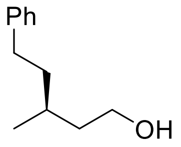

2f-OH

racemic

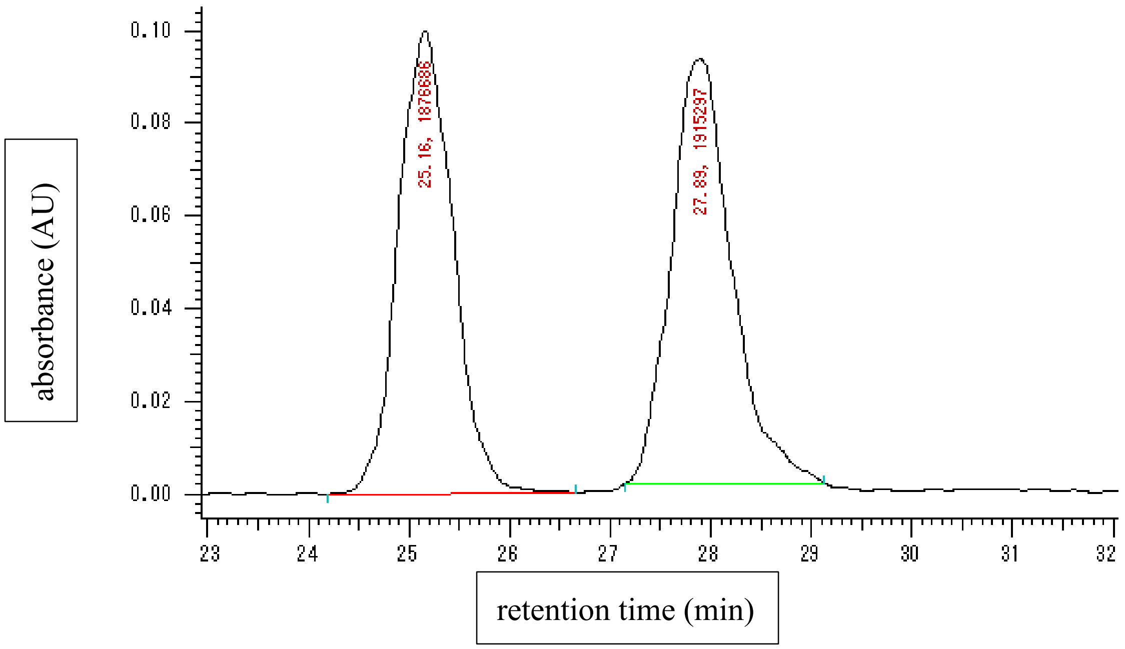

chiral

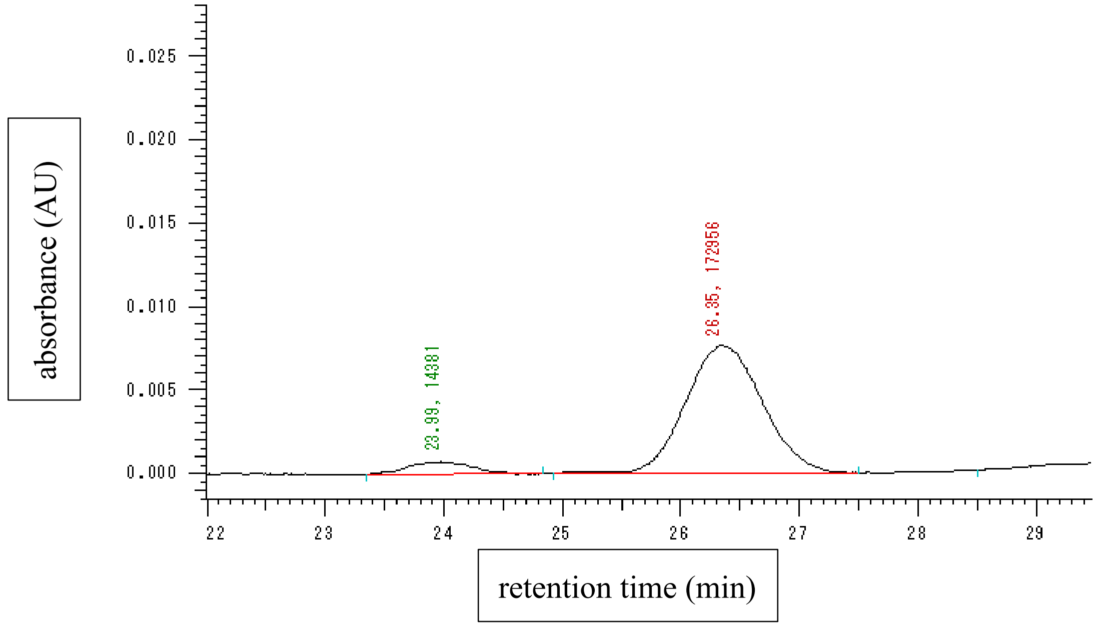

racemic | chiral

no.	RT (min)	area	area (%)	no.	RT (min)	area	area (%)
1	25.16	1876846	48.220	1	23.99	14381	7.677
2	27.89	2015415	51.780	2	26.35	172956	92.323

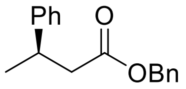

2g

racemic

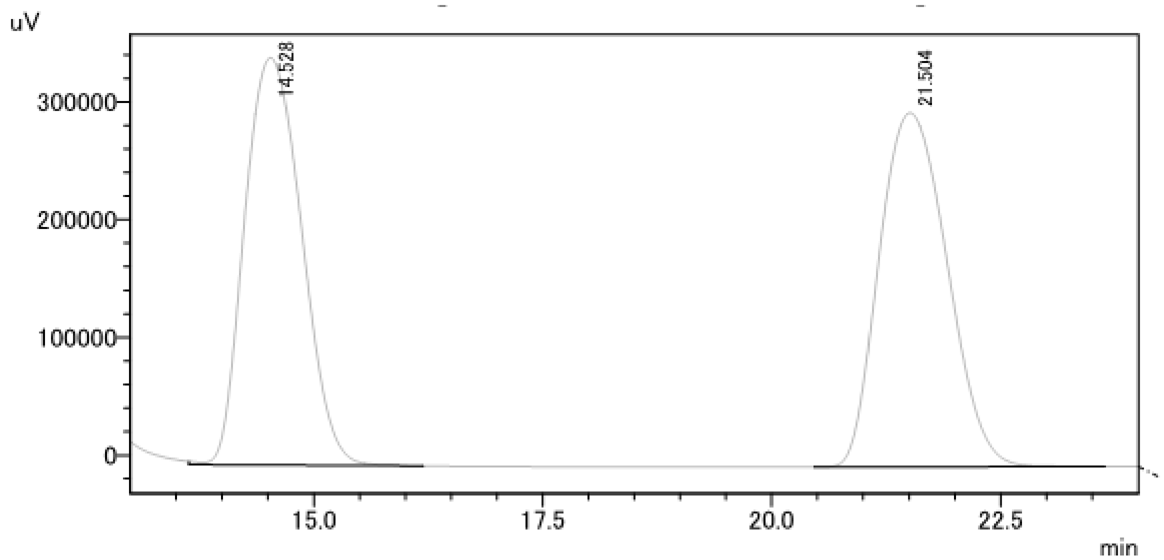

chiral

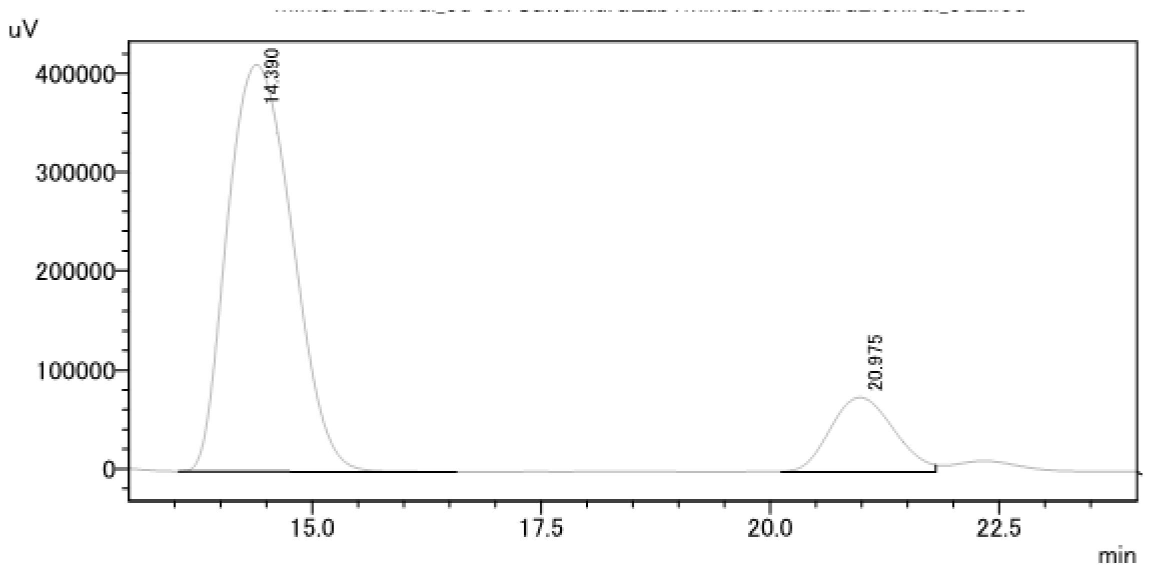

racemic				chiral			
no.	RT (min)	area	area (%)	no.	RT (min)	area	area (%)
1	14.528	14901109	49.020	1	14.390	20309892	84.832
2	21.504	15496605	50.980	2	20.975	3631436	15.168

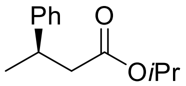

2h

racemic

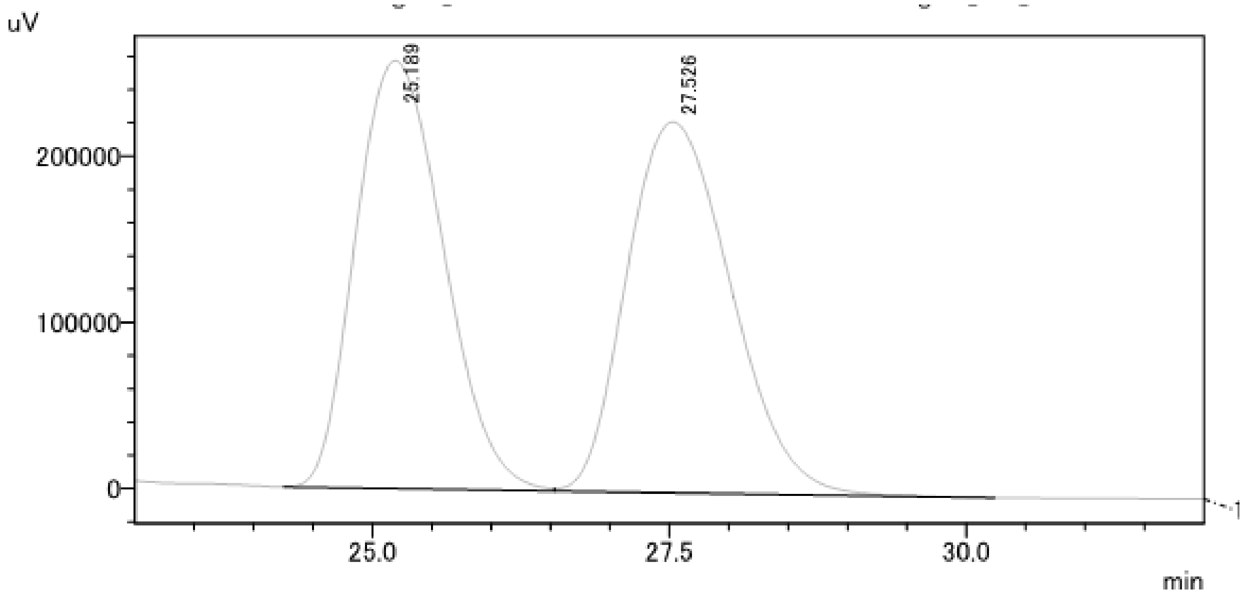

chiral

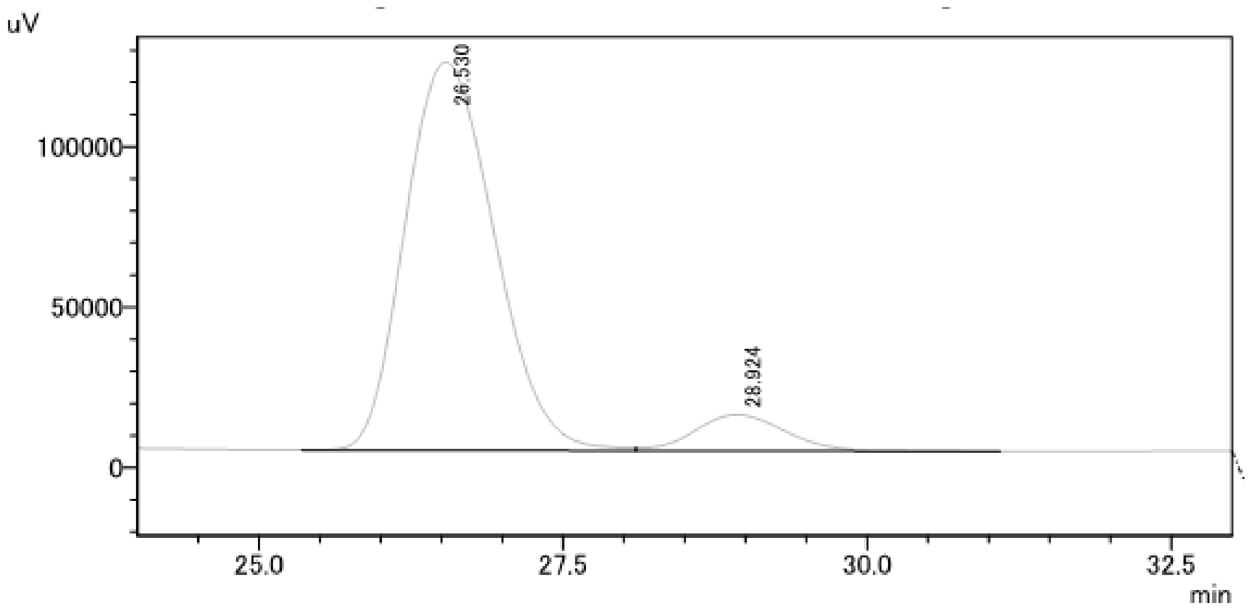

racemic				chiral			
no.	RT (min)	area	area (%)	no.	RT (min)	area	area (%)
1	25.189	13240552	49.281	1	26.530	6169064	91.304
2	27.526	13627152	50.719	2	28.924	587574	8.696

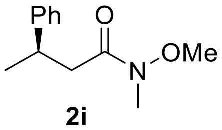

2i

racemic

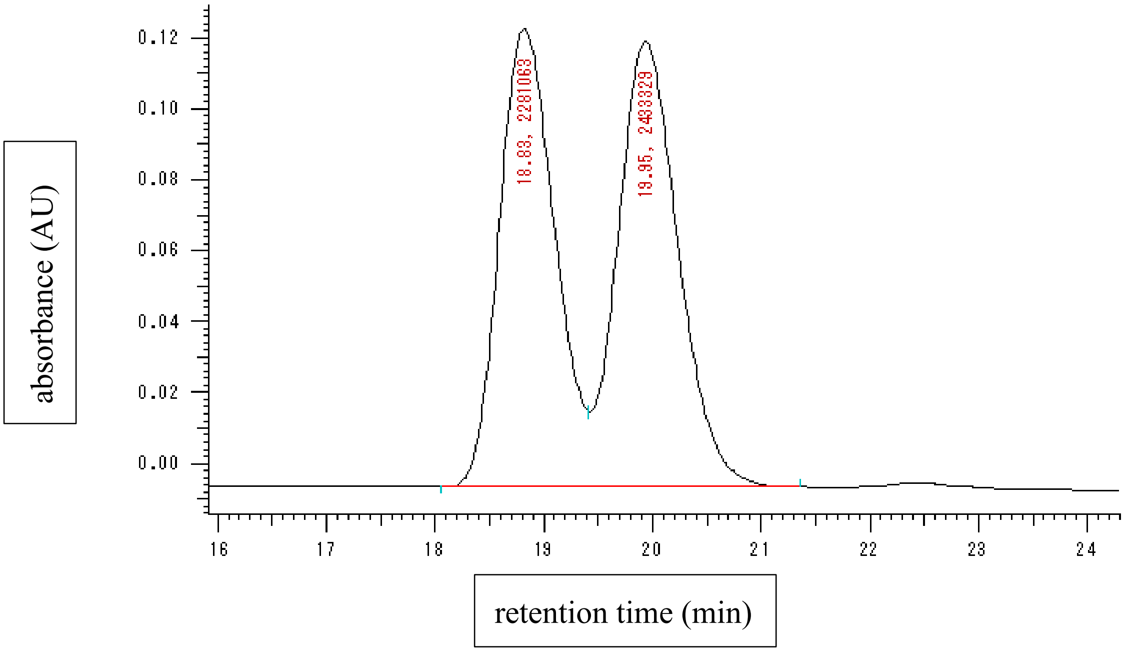

chiral

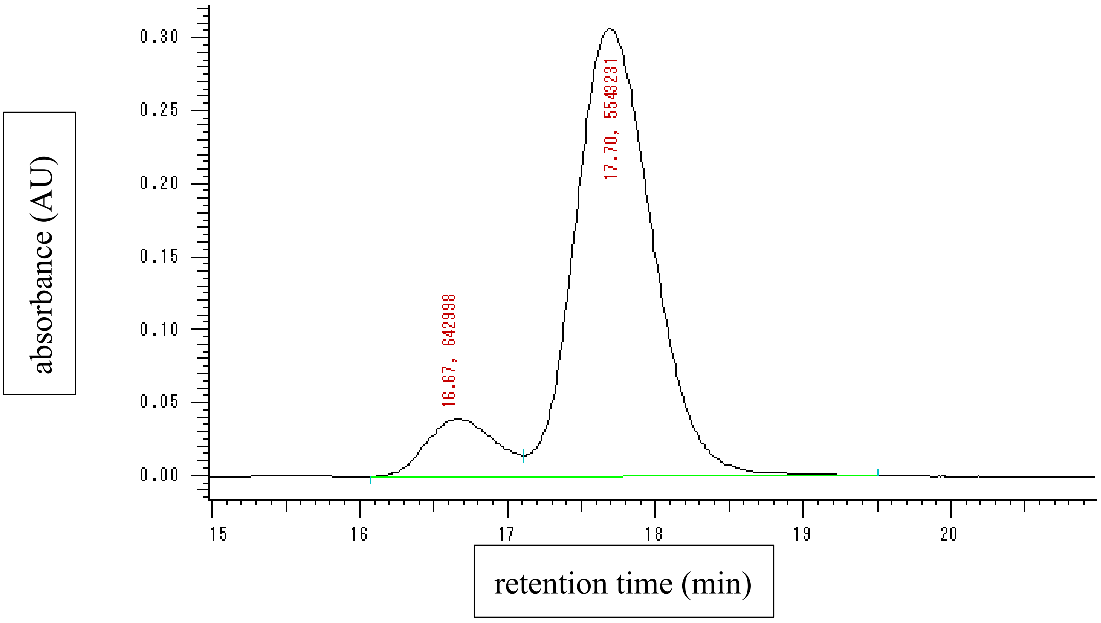

racemic				chiral			
no.	RT (min)	area	area (%)	no.	RT (min)	area	area (%)
1	18.83	2281063	48.385	1	16.67	642998	10.394
2	19.95	2433329	51.615	2	17.70	5543231	89.606

NMR Spectra

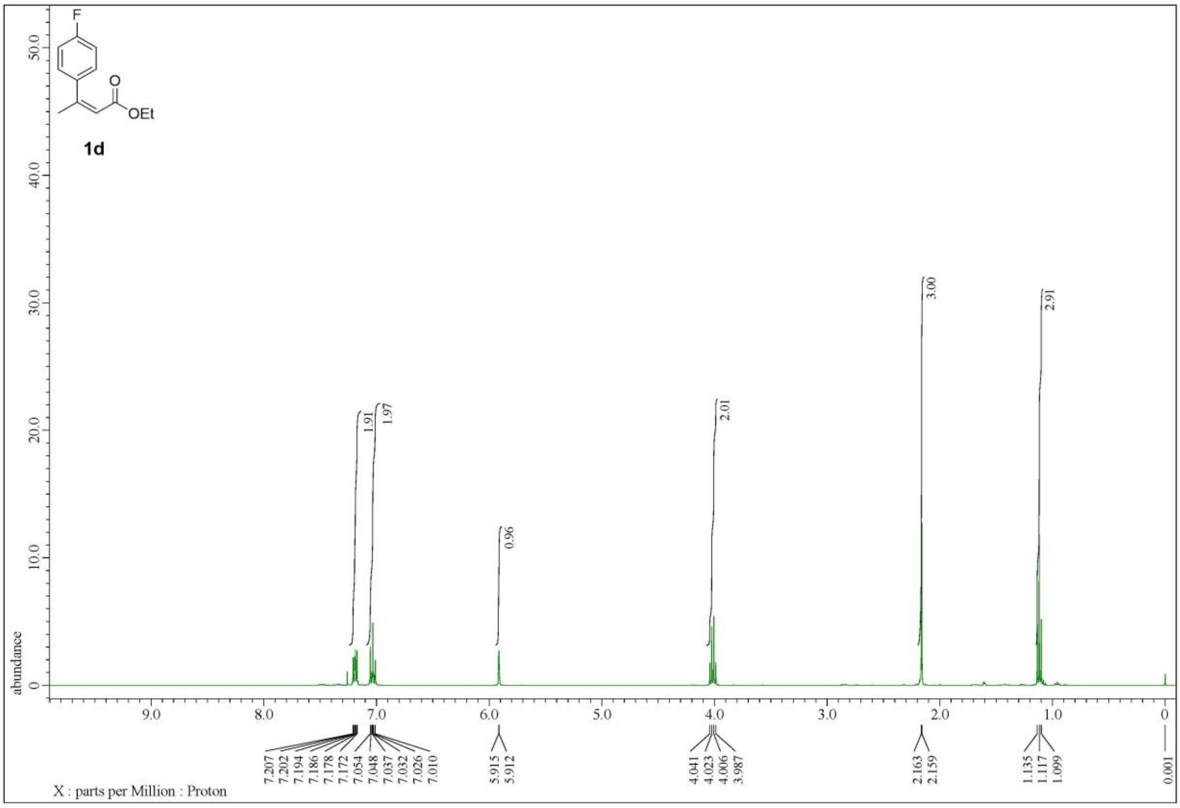

F
O
OEt
1d
abundance
X : parts per Million : Proton

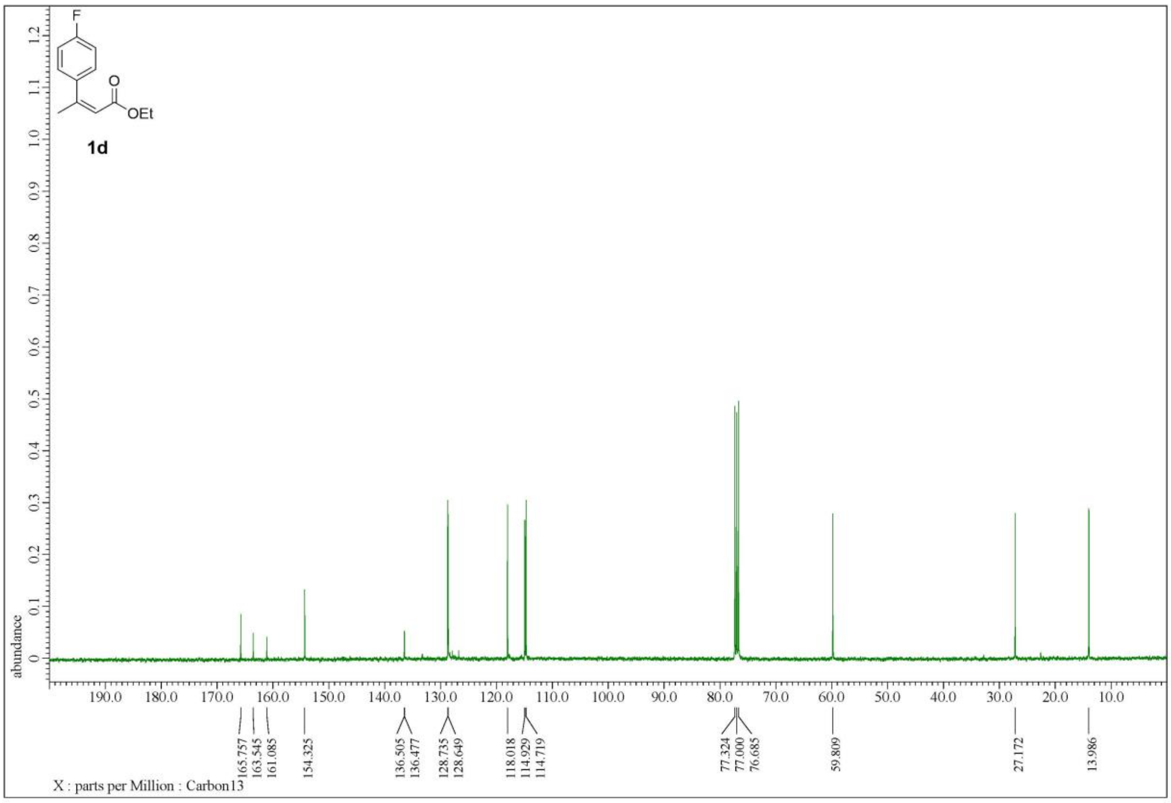

F
O
OEt
1d
abundance
X : parts per Million : Carbon13

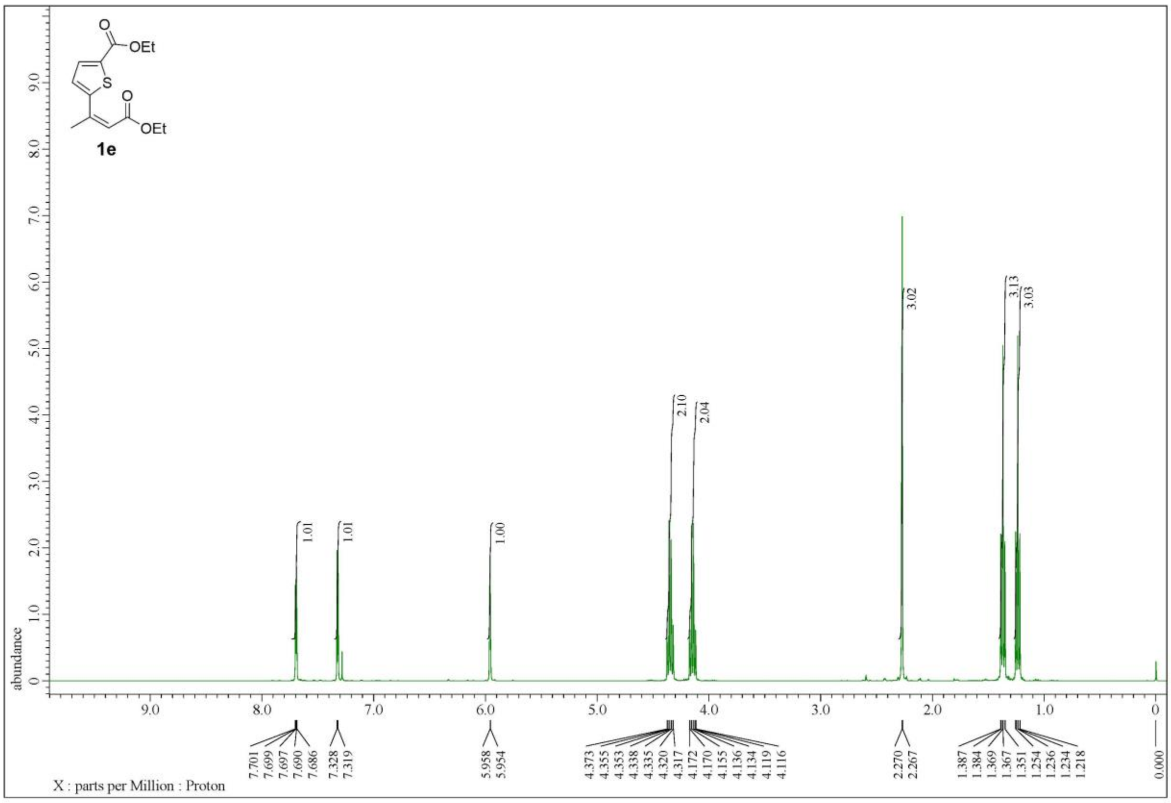
OEt
S
OEt
1e
abundance
X : parts per Million : Proton
1.01
1.01
1.00
2.10
2.04
3.02
3.13
3.03
7.701
7.699
7.697
7.690
7.686
7.328
7.319
5.958
5.954
4.373
4.355
4.353
4.338
4.335
4.320
4.317
4.172
4.170
4.155
4.136
4.134
4.119
4.116
2.270
2.267
1.387
1.384
1.369
1.367
1.351
1.254
1.236
1.234
1.218
0.000

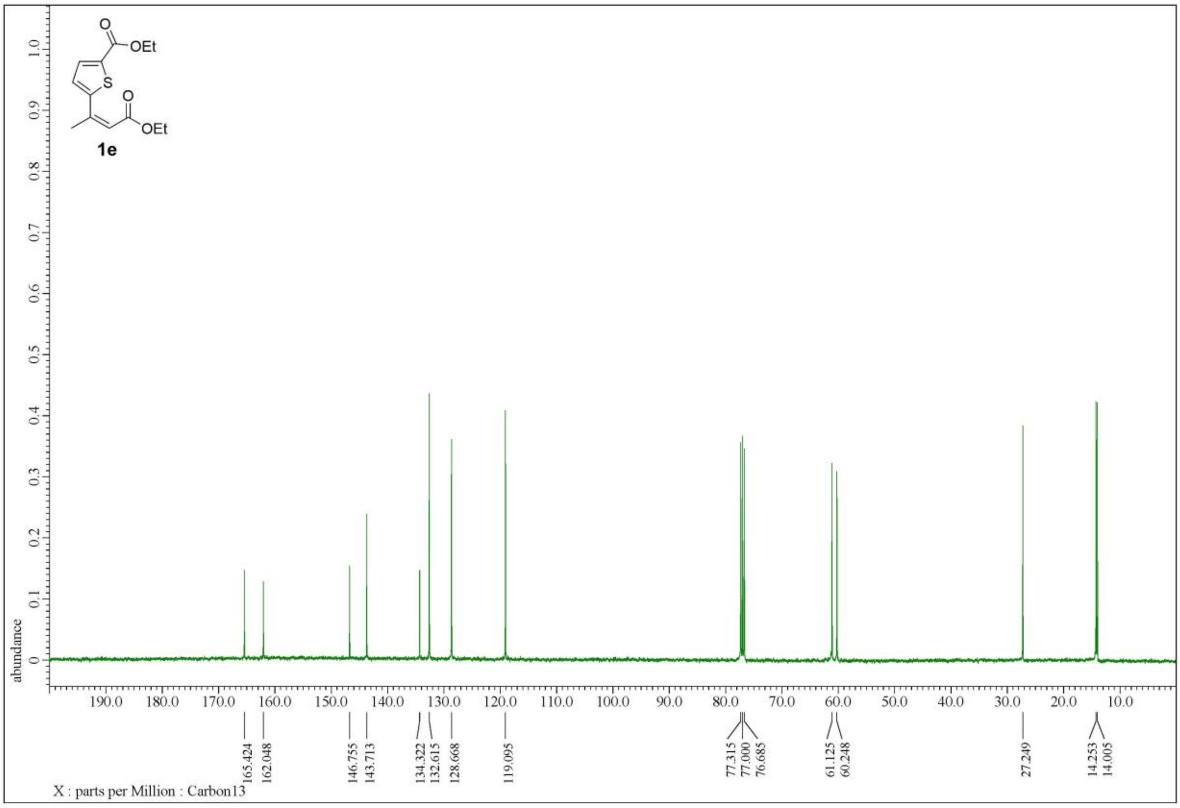
OEt
S
OEt
1e
abundance
X : parts per Million : Carbon13
165.424
162.048
146.755
143.713
134.322
132.615
128.668
119.095
77.315
77.000
76.685
61.125
60.248
27.249
14.253
14.005

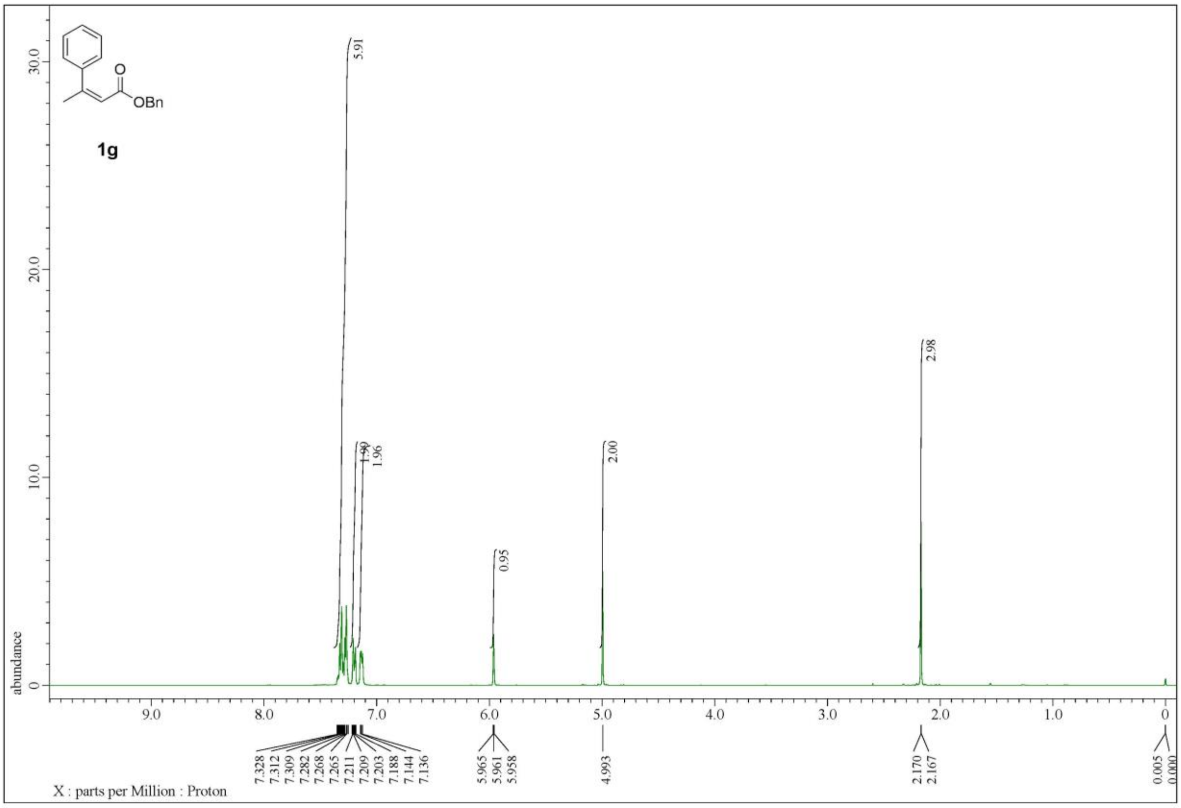

OBn
1g
abundance
X : parts per Million : Proton
5.91
1.90
1.96
0.95
2.00
2.98
7.328
7.312
7.309
7.282
7.268
7.265
7.211
7.209
7.203
7.188
7.144
7.136
5.965
5.961
5.958
4.993
2.170
2.167
0.005
0.000

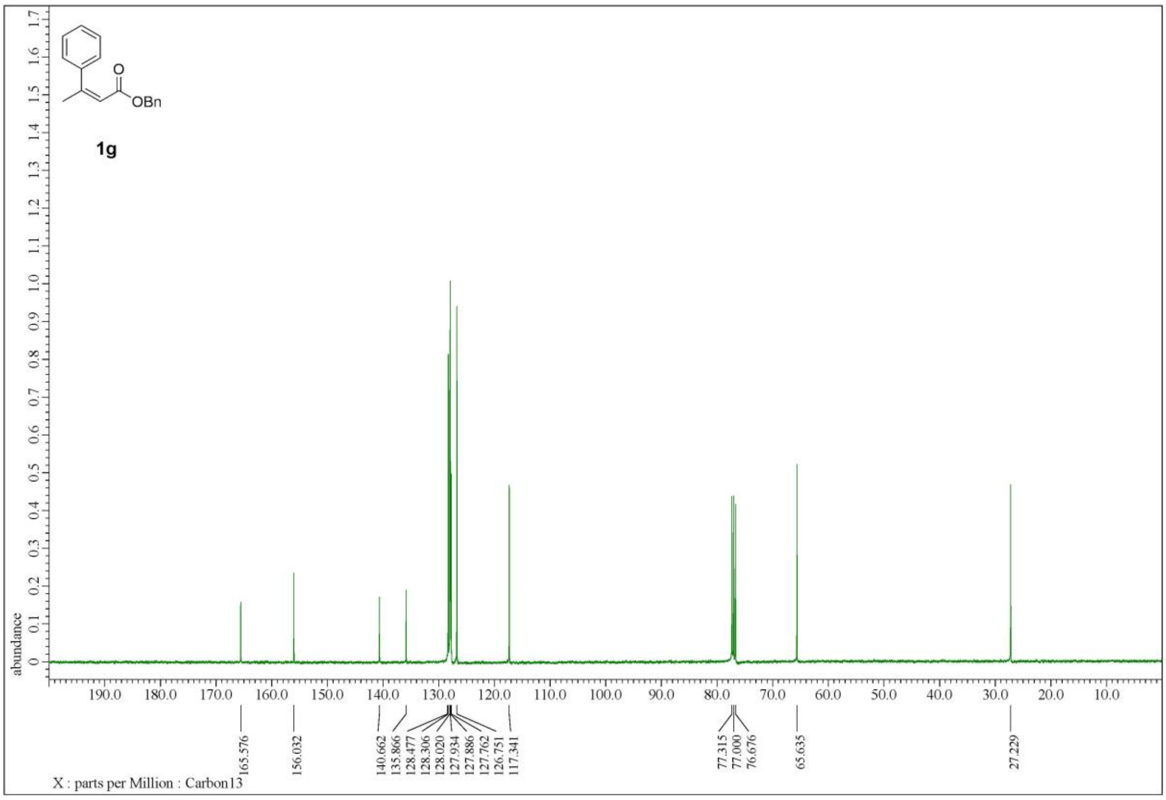

OBn
1g
abundance
X : parts per Million : Carbon13
165.576
156.032
140.662
135.866
128.477
128.306
128.020
127.934
127.886
127.762
126.751
117.341
77.315
77.000
76.676
65.635
27.229

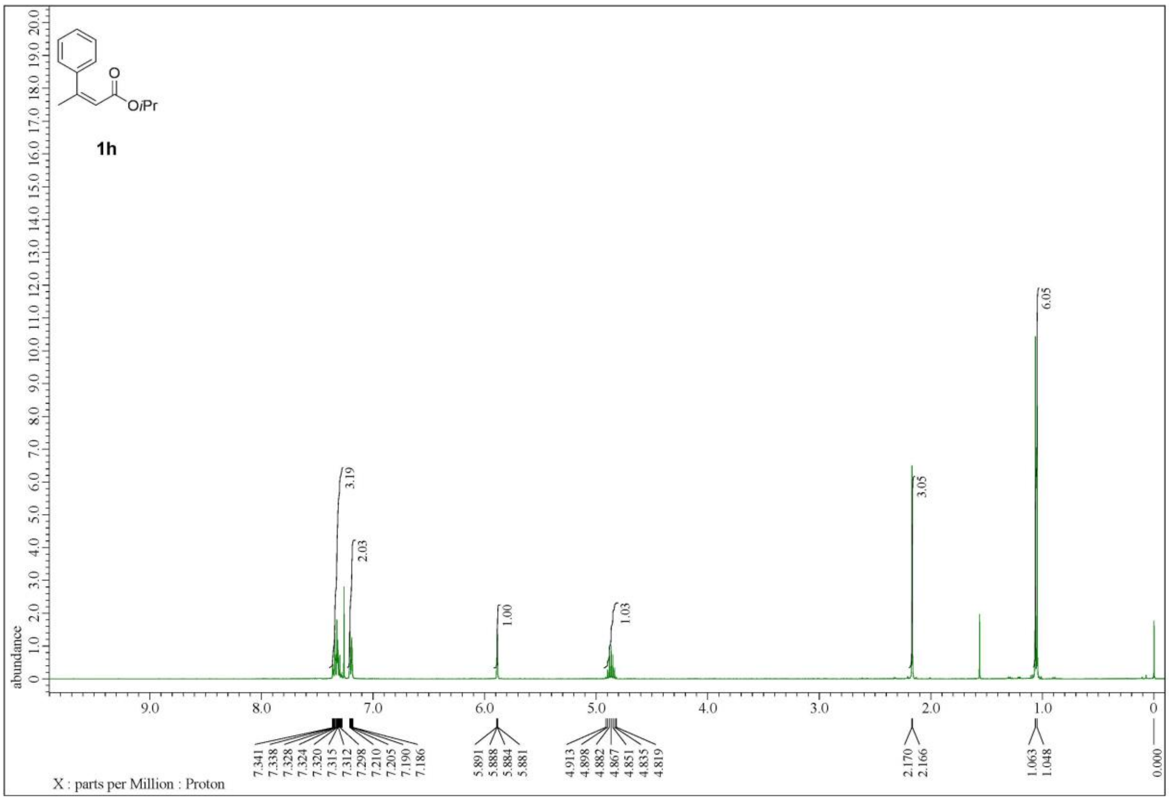
1h
O*i*Pr
abundance
X : parts per Million : Proton
3.19
2.03
1.00
1.03
3.05
6.05
7.341 7.338 7.328 7.324 7.320 7.315 7.312 7.298 7.210 7.205 7.190 7.186
5.891 5.888 5.884 5.881
4.913 4.898 4.882 4.867 4.851 4.835 4.819
2.170 2.166
1.063 1.048
0.000

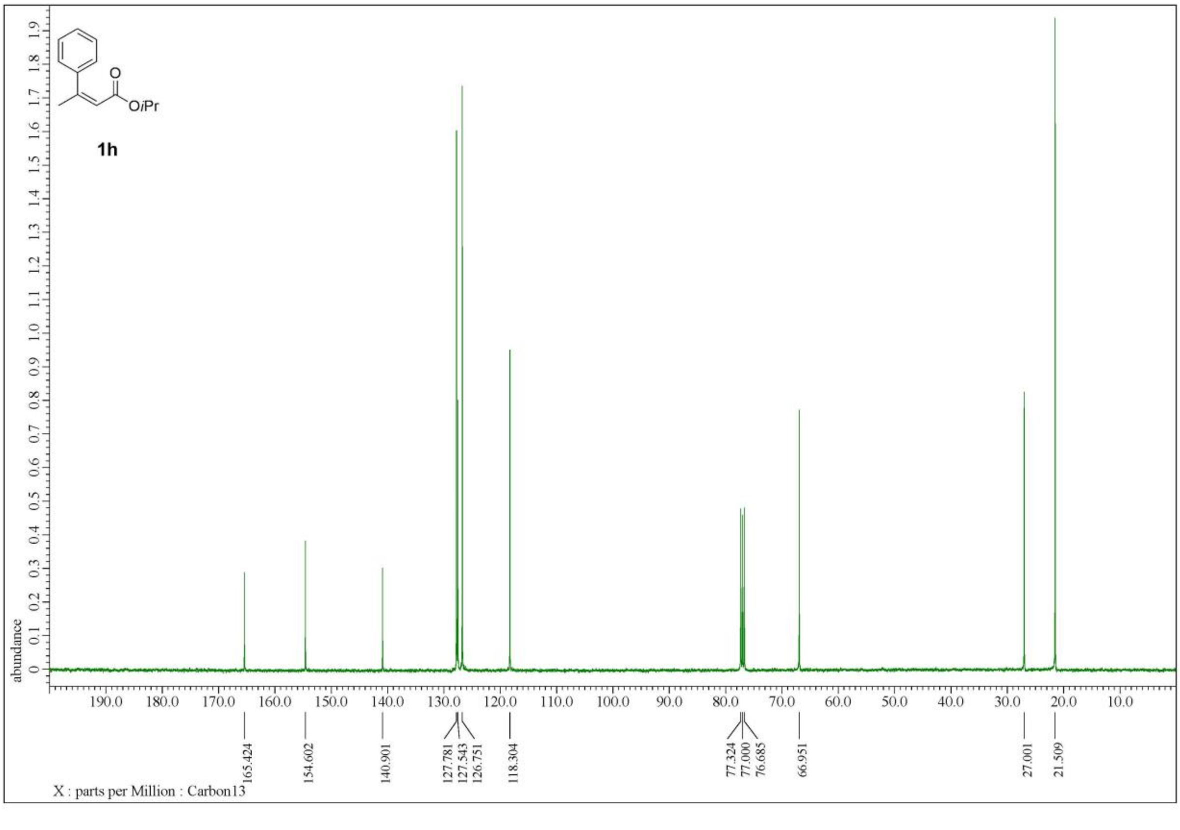
1h
O*i*Pr
abundance
X : parts per Million : Carbon13
165.424
154.602
140.901
127.781 127.543 126.751
118.304
77.324 77.000 76.685
66.951
27.001
21.509

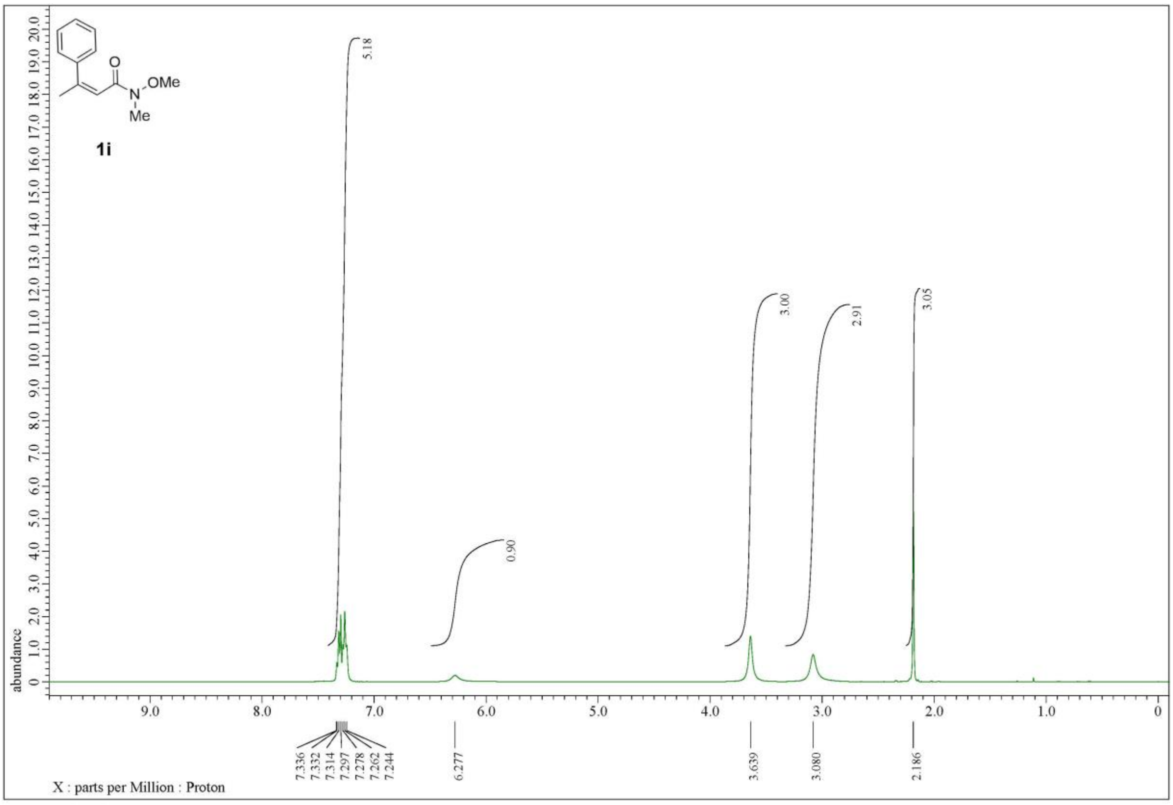
OMe
N
Me
O
1i
abundance
5.18
0.90
3.00
2.91
3.05
9.0
8.0
7.0
6.0
5.0
4.0
3.0
2.0
1.0
0
7.336
7.332
7.314
7.297
7.278
7.262
7.244
6.277
3.639
3.080
2.186
X : parts per Million : Proton

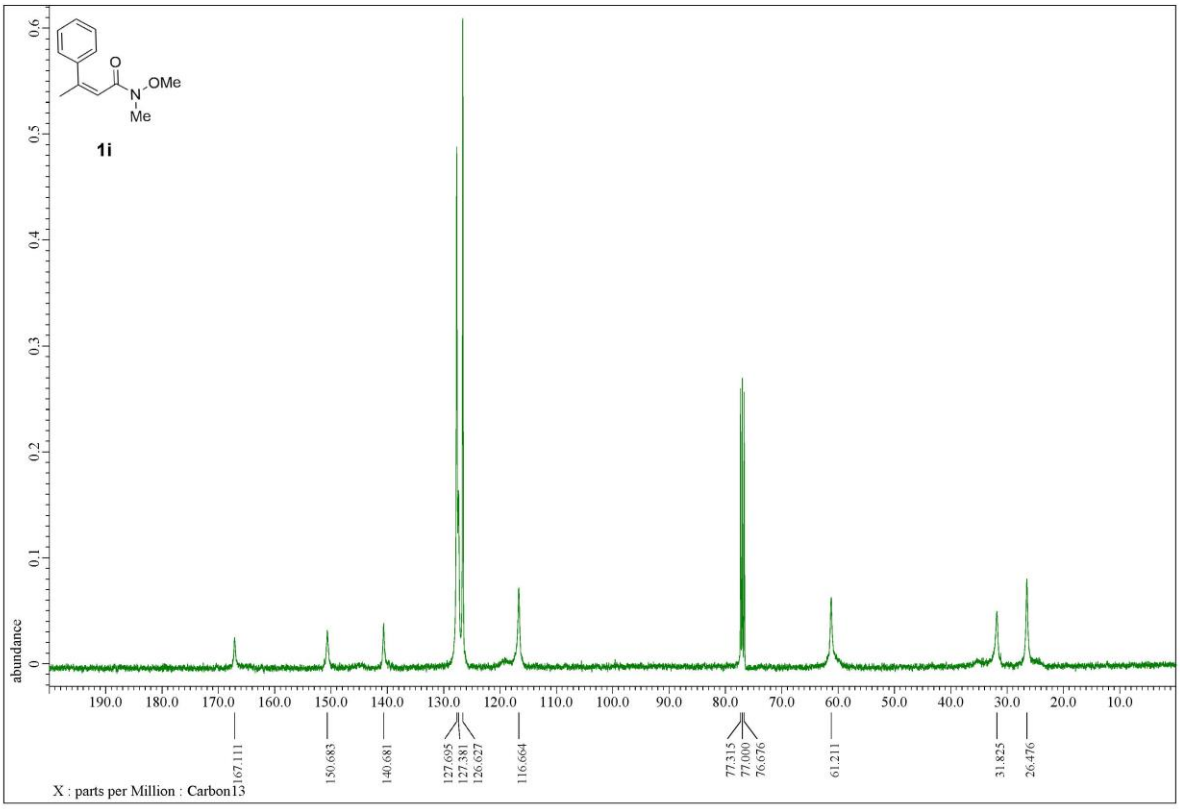
OMe
N
Me
O
1i
abundance
190.0
180.0
170.0
160.0
150.0
140.0
130.0
120.0
110.0
100.0
90.0
80.0
70.0
60.0
50.0
40.0
30.0
20.0
10.0
167.111
150.683
140.681
127.695
127.381
126.627
116.664
77.315
77.000
76.676
61.211
31.825
26.476
X : parts per Million : Carbon13

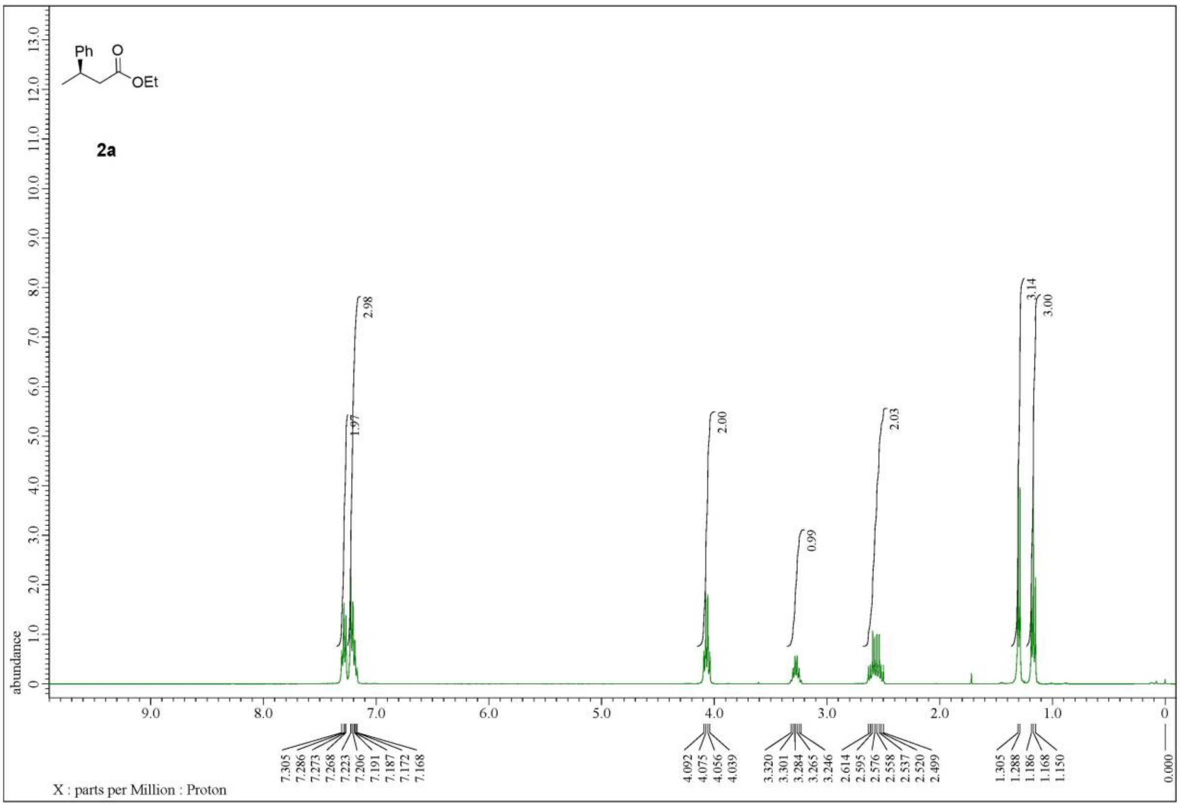
Ph
O
OEt
2a
abundance
X : parts per Million : Proton
2.98
1.97
2.00
0.99
2.03
3.14
3.00
7.305
7.286
7.273
7.268
7.223
7.206
7.191
7.187
7.172
7.168
4.092
4.075
4.056
4.039
3.320
3.301
3.284
3.265
3.246
2.614
2.595
2.576
2.558
2.537
2.520
2.499
1.305
1.288
1.186
1.168
1.150
0.000

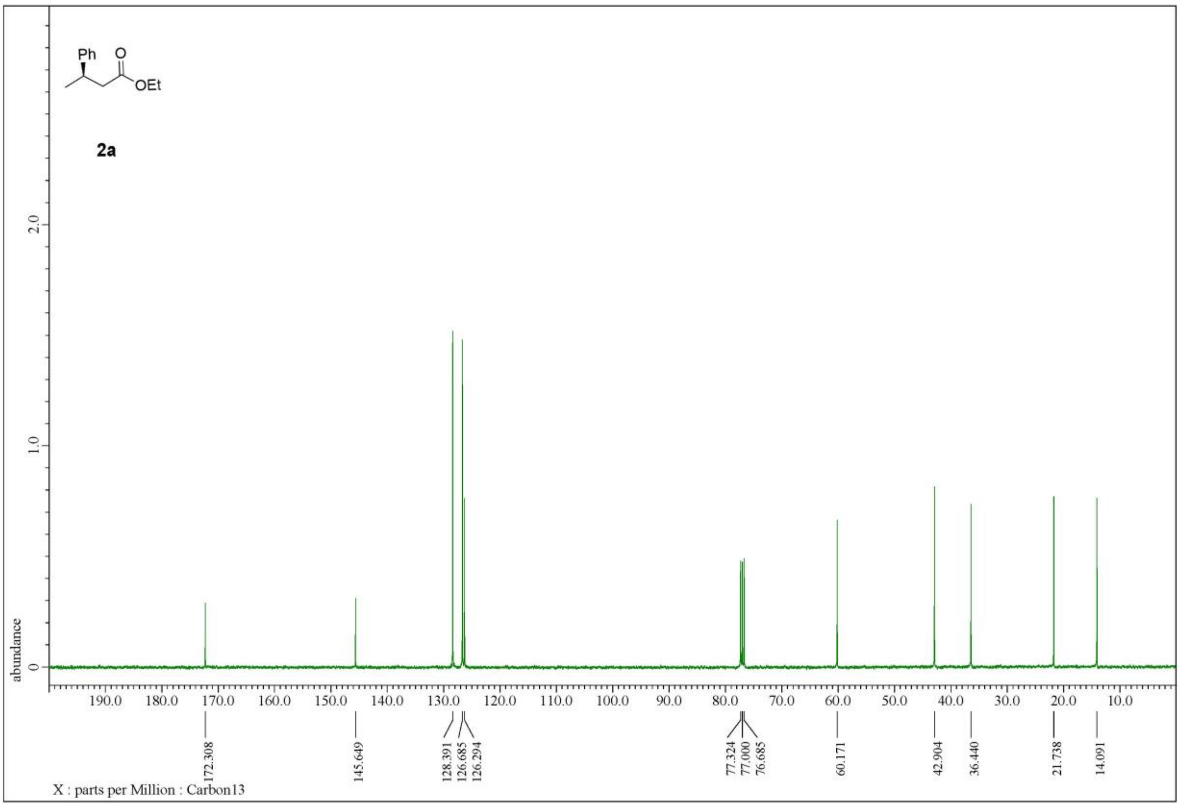
Ph
O
OEt
2a
abundance
X : parts per Million : Carbon13
172.308
145.649
128.391
126.685
126.294
77.324
77.000
76.685
60.171
42.904
36.440
21.738
14.091

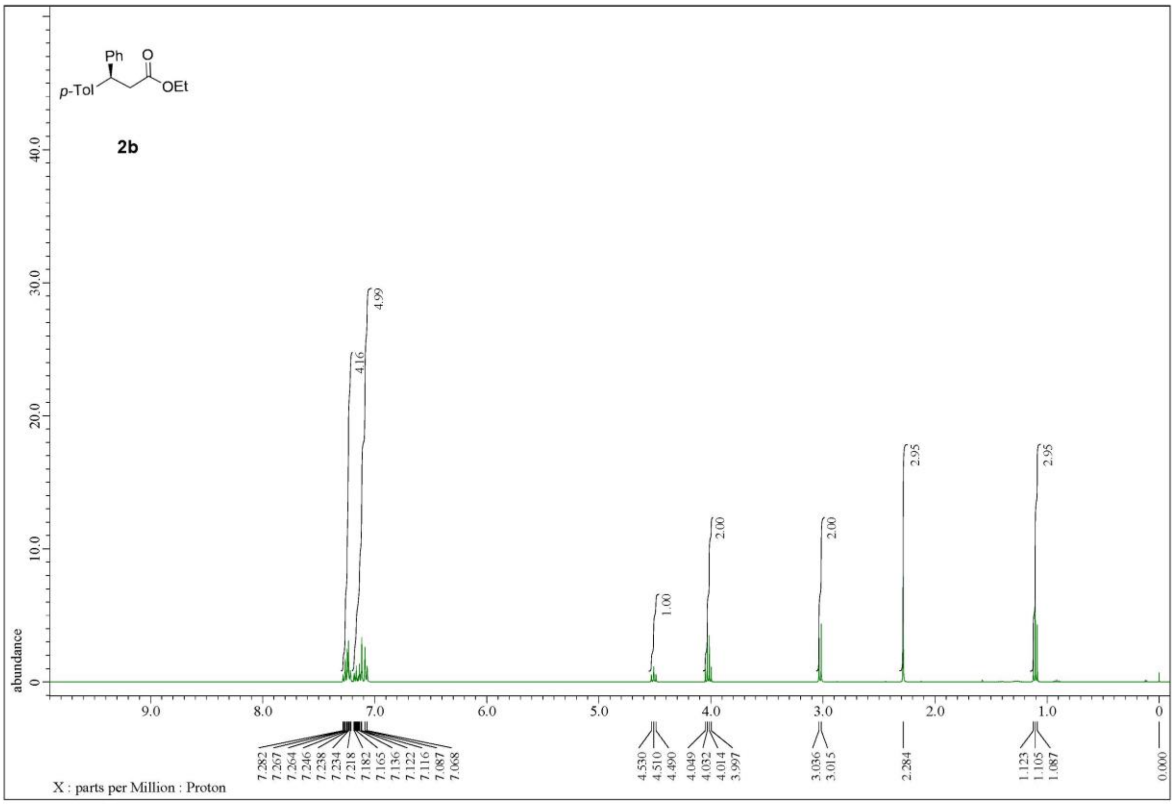
Ph
O
p-Tol
OEt
2b
abundance
X : parts per Million : Proton
7.282
7.267
7.264
7.246
7.238
7.234
7.218
7.182
7.165
7.136
7.122
7.116
7.087
7.068
4.530
4.510
4.490
4.049
4.032
4.014
3.997
3.036
3.015
2.284
1.123
1.105
1.087
0.000
4.16
4.99
1.00
2.00
2.00
2.95
2.95

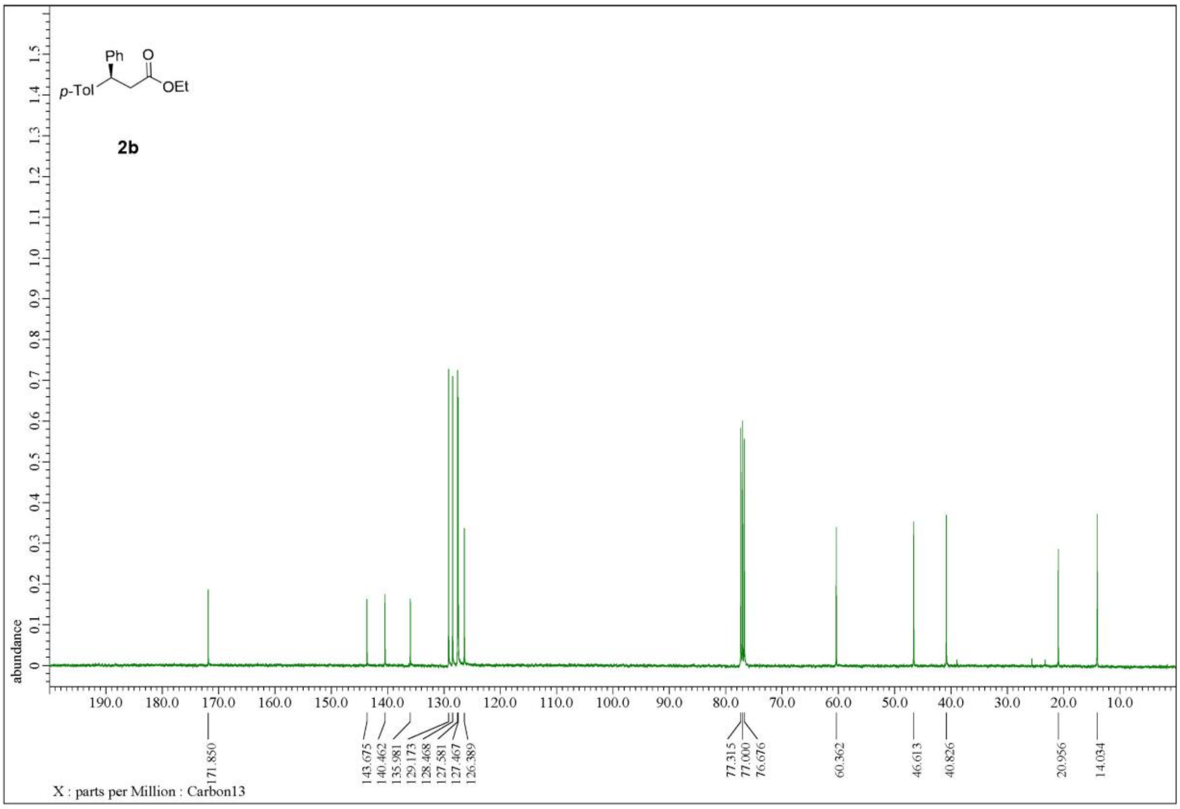
Ph
O
p-Tol
OEt
2b
abundance
X : parts per Million : Carbon13
171.850
143.675
140.462
135.981
129.173
128.468
127.581
127.467
126.389
77.315
77.000
76.676
60.362
46.613
40.826
20.956
14.034

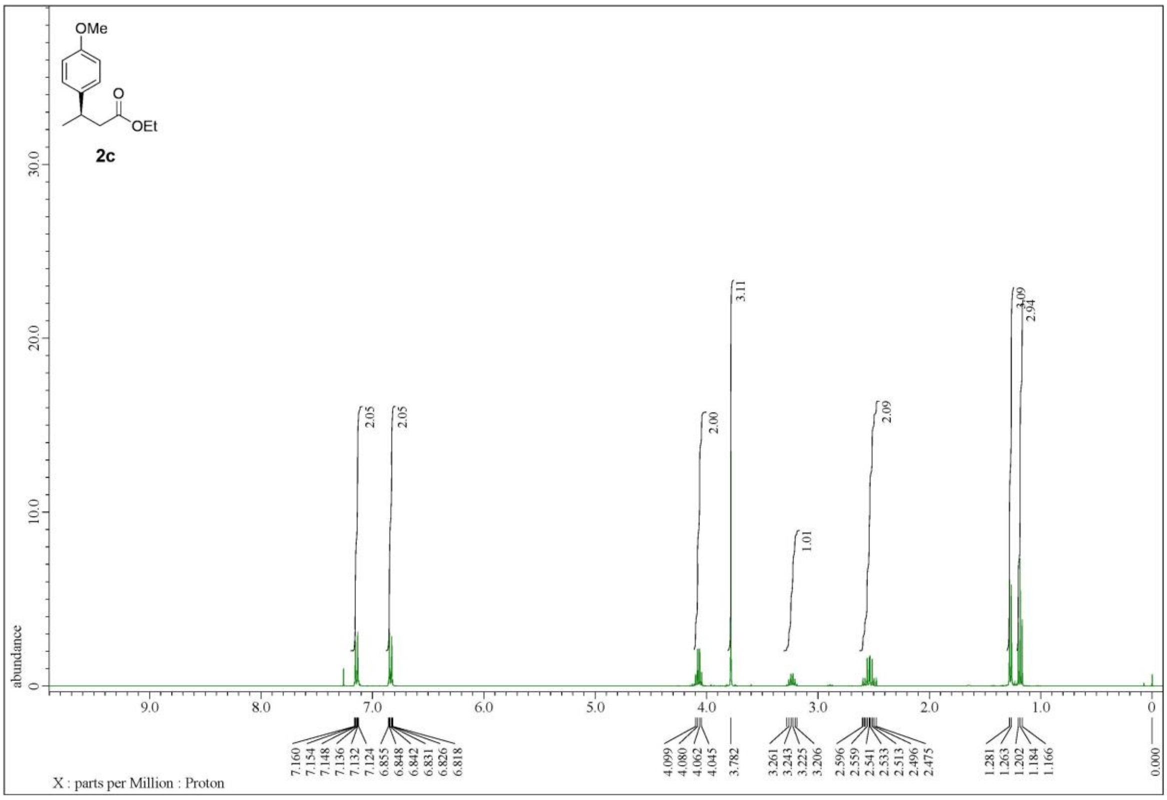
OMe
O
OEt
2c
abundance
X : parts per Million : Proton
3.11
3.09
2.94
2.05
2.05
2.00
2.09
1.01
7.160
7.154
7.148
7.136
7.132
7.124
6.855
6.848
6.842
6.831
6.826
6.818
4.099
4.080
4.062
4.045
3.782
3.261
3.243
3.225
3.206
2.596
2.559
2.541
2.533
2.513
2.496
2.475
1.281
1.263
1.202
1.184
1.166
0.000

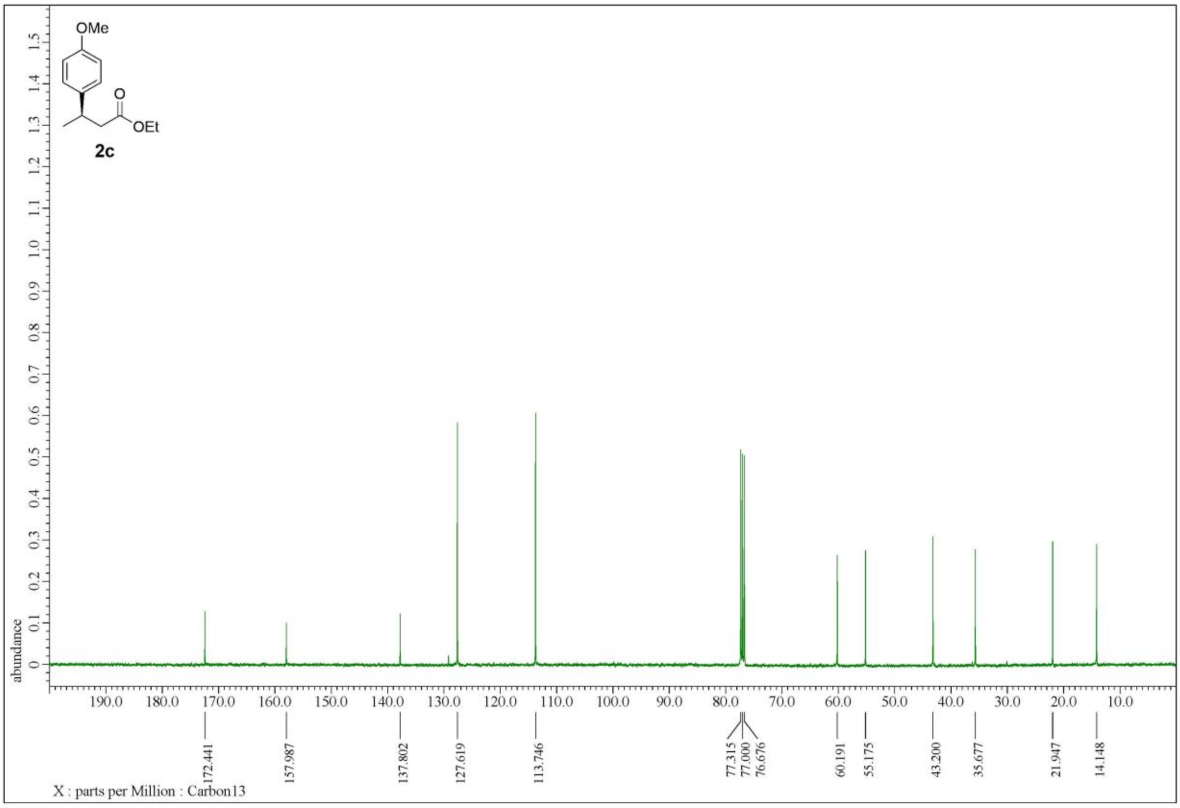
OMe
O
OEt
2c
abundance
X : parts per Million : Carbon13
172.441
157.987
137.802
127.619
113.746
77.315
77.000
76.676
60.191
55.175
43.200
35.677
21.947
14.148

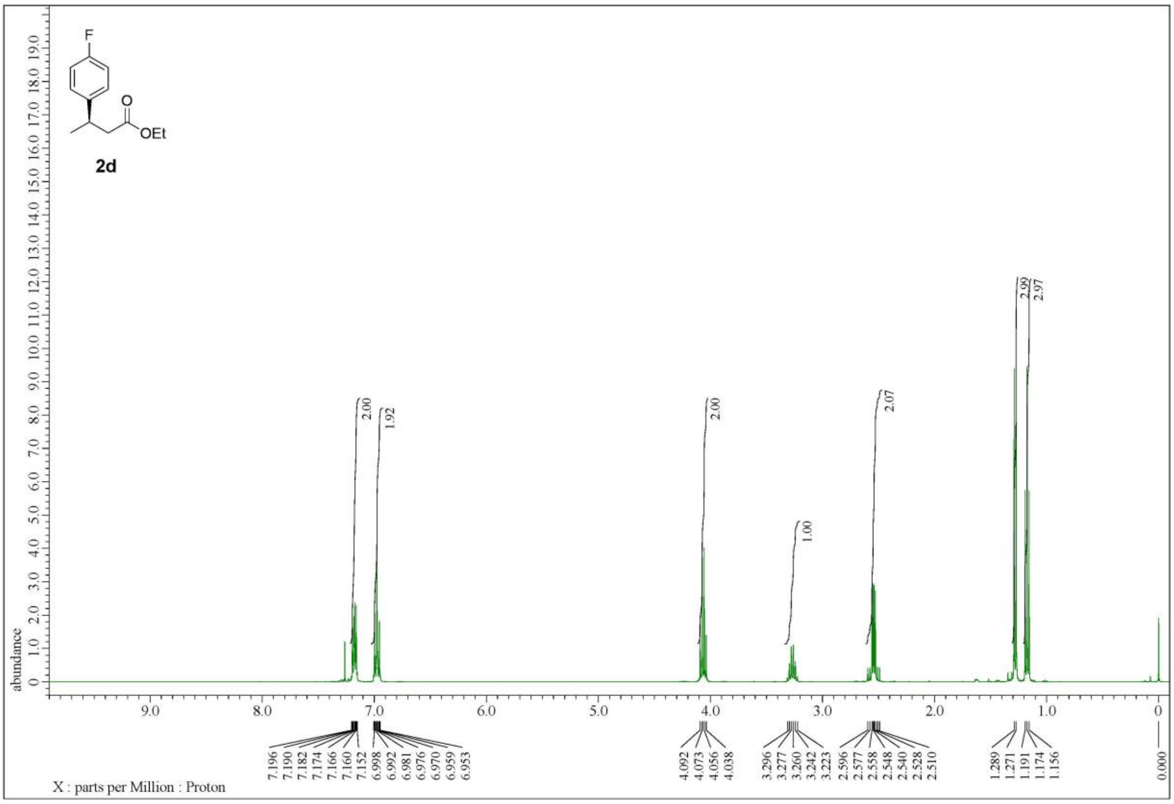
F
O
OEt
2d
abundance
2.00
1.92
2.00
1.00
2.07
2.99
2.97
7.196
7.190
7.182
7.174
7.166
7.160
7.152
6.998
6.992
6.981
6.976
6.970
6.959
6.953
4.092
4.073
4.056
4.038
3.296
3.277
3.260
3.242
3.223
2.596
2.577
2.558
2.548
2.540
2.528
2.510
1.289
1.271
1.191
1.174
1.156
0.000
X : parts per Million : Proton

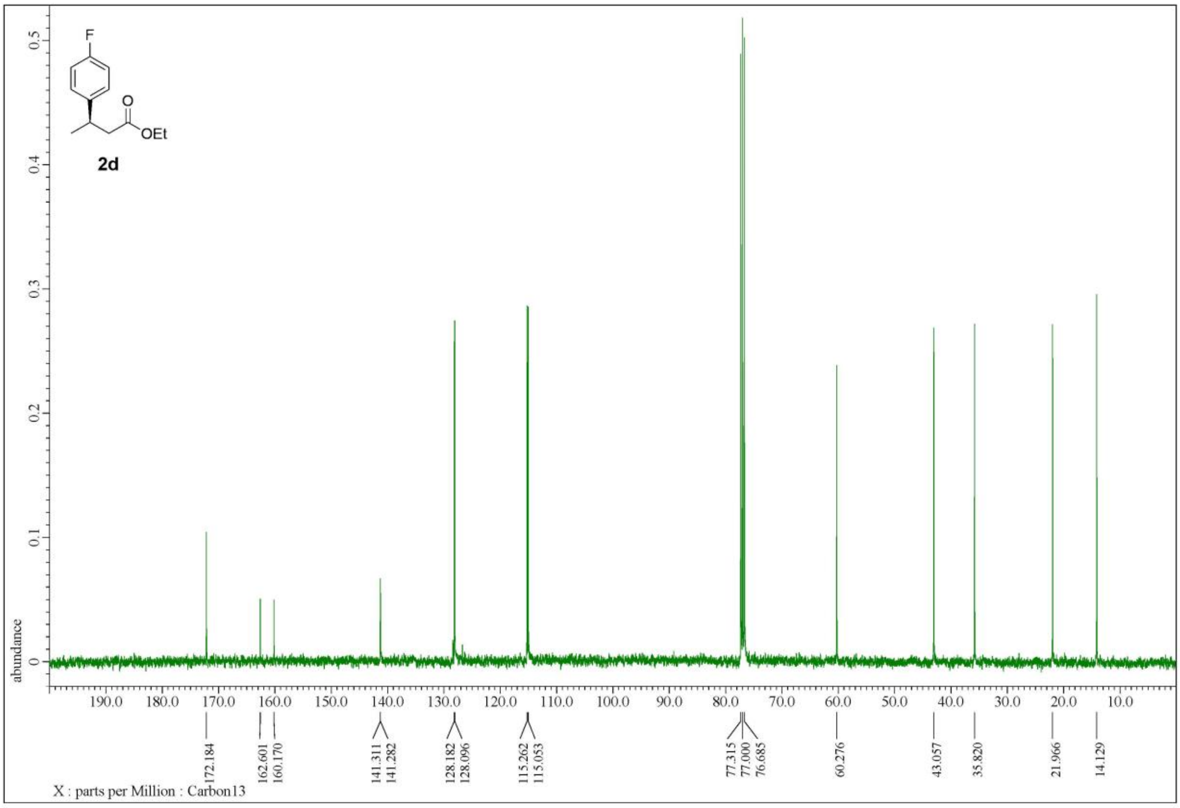
F
O
OEt
2d
abundance
172.184
162.601
160.170
141.311
141.282
128.182
128.096
115.262
115.053
77.315
77.000
76.685
60.276
43.057
35.820
21.966
14.129
X : parts per Million : Carbon13

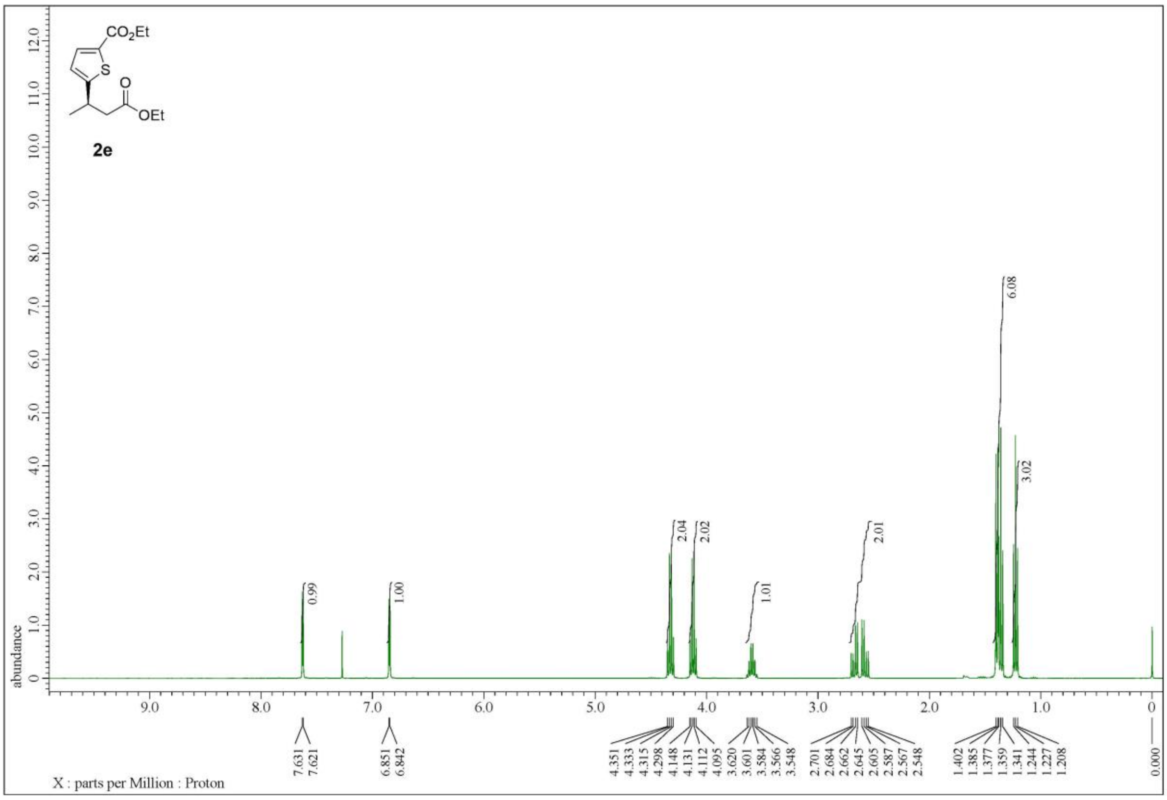
CO2Et
S
O
OEt
2e
abundance
X : parts per Million : Proton
0.99
1.00
2.04
2.02
1.01
2.01
6.08
3.02
7.631
7.621
6.851
6.842
4.351
4.333
4.315
4.298
4.148
4.131
4.112
4.095
3.620
3.601
3.584
3.566
3.548
2.701
2.684
2.662
2.645
2.605
2.587
2.567
2.548
1.402
1.385
1.377
1.359
1.341
1.244
1.227
1.208
0.000

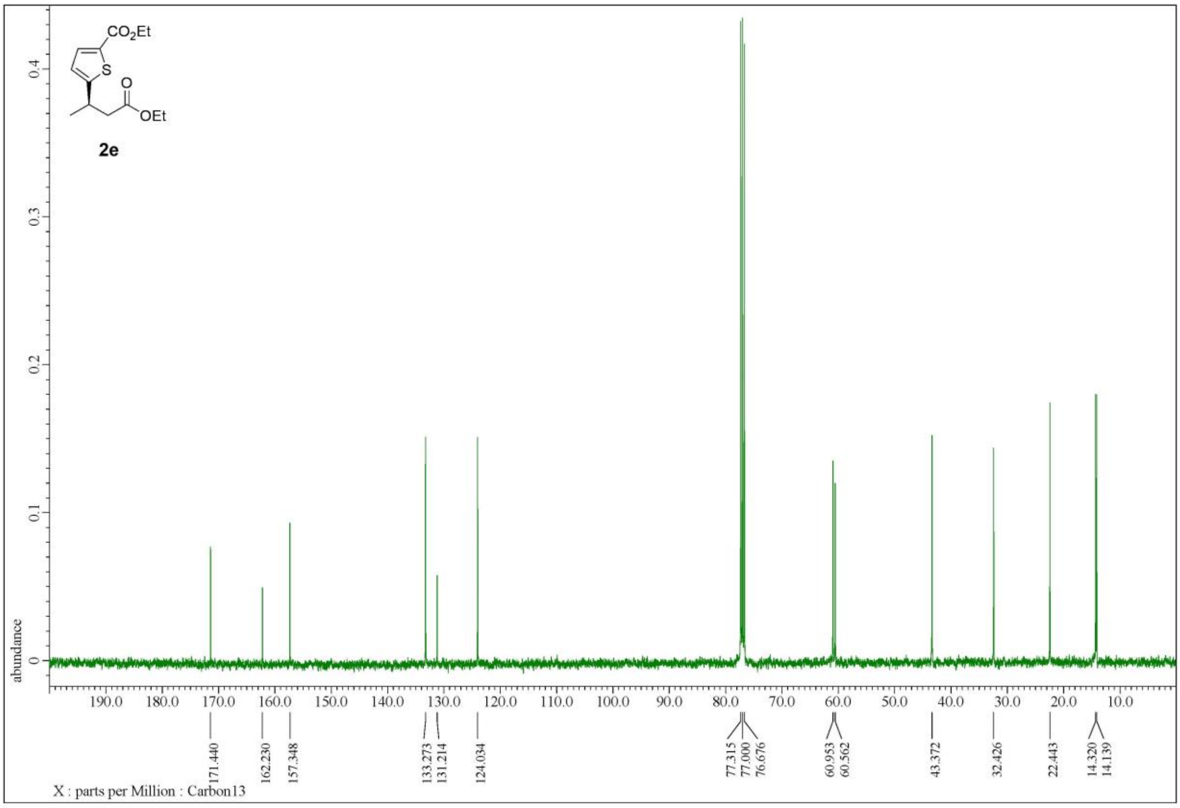
CO2Et
S
O
OEt
2e
abundance
X : parts per Million : Carbon13
171.440
162.230
157.348
133.273
131.214
124.034
77.315
77.000
76.676
60.953
60.562
43.372
32.426
22.443
14.320
14.139

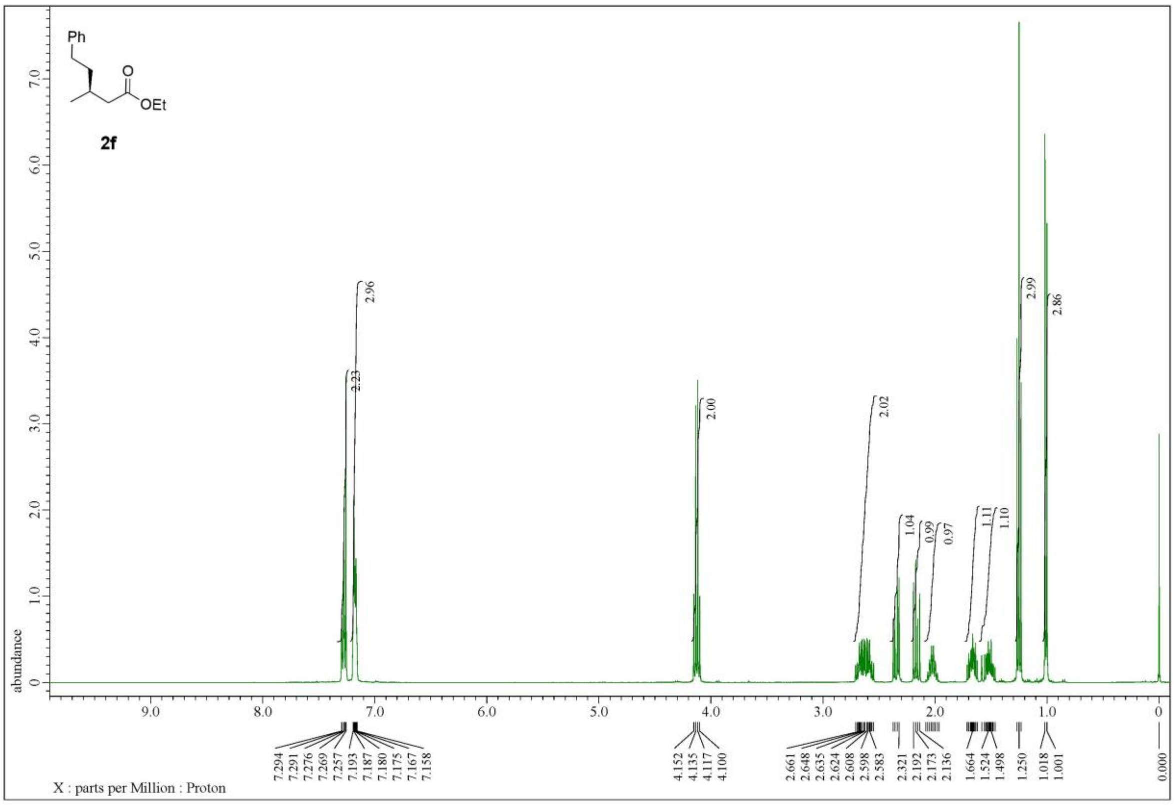
Ph
O
OEt
2f
abundance
X : parts per Million : Proton
2.96
2.23
2.00
2.02
1.04
0.99
0.97
1.11
1.10
2.99
2.86
7.294
7.291
7.276
7.269
7.257
7.193
7.187
7.180
7.175
7.167
7.158
4.152
4.135
4.117
4.100
2.661
2.648
2.635
2.624
2.608
2.598
2.583
2.321
2.192
2.173
2.136
1.664
1.524
1.498
1.250
1.018
1.001
0.000

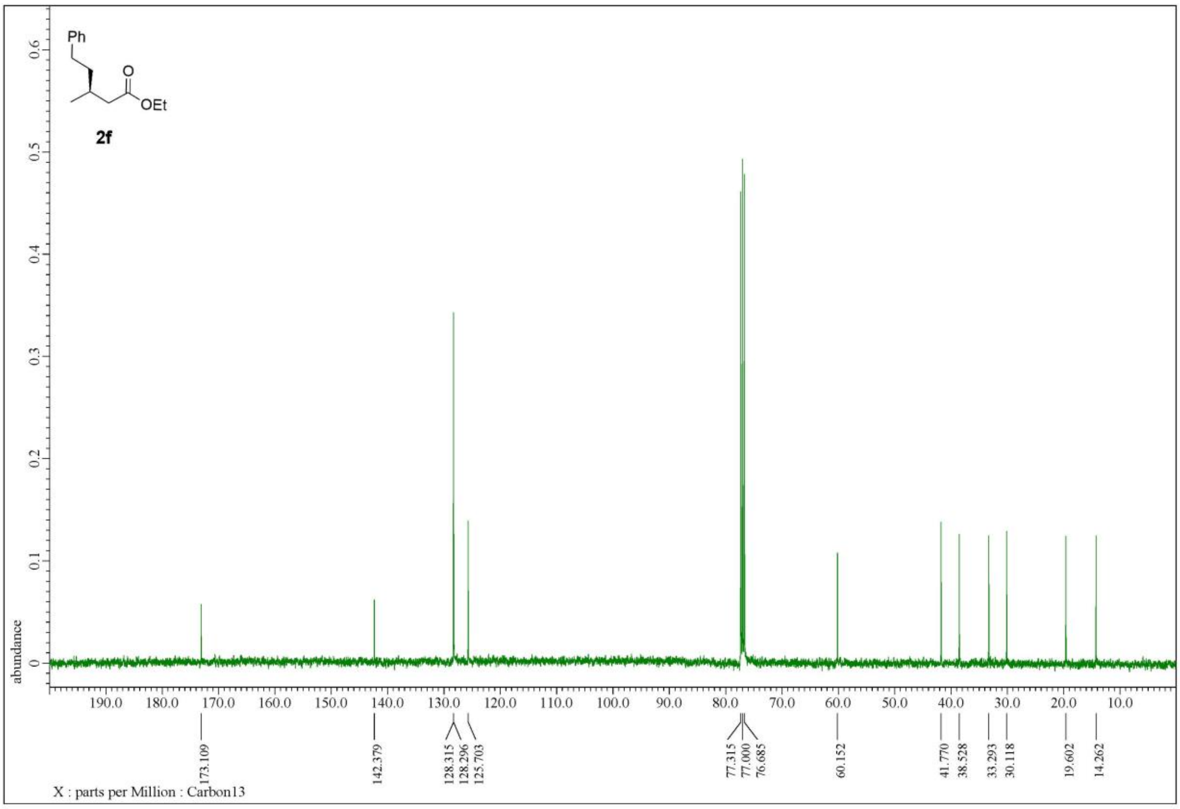
Ph
O
OEt
2f
abundance
X : parts per Million : Carbon13
173.109
142.379
128.315
128.296
125.703
77.315
77.000
76.685
60.152
41.770
38.528
33.293
30.118
19.602
14.262

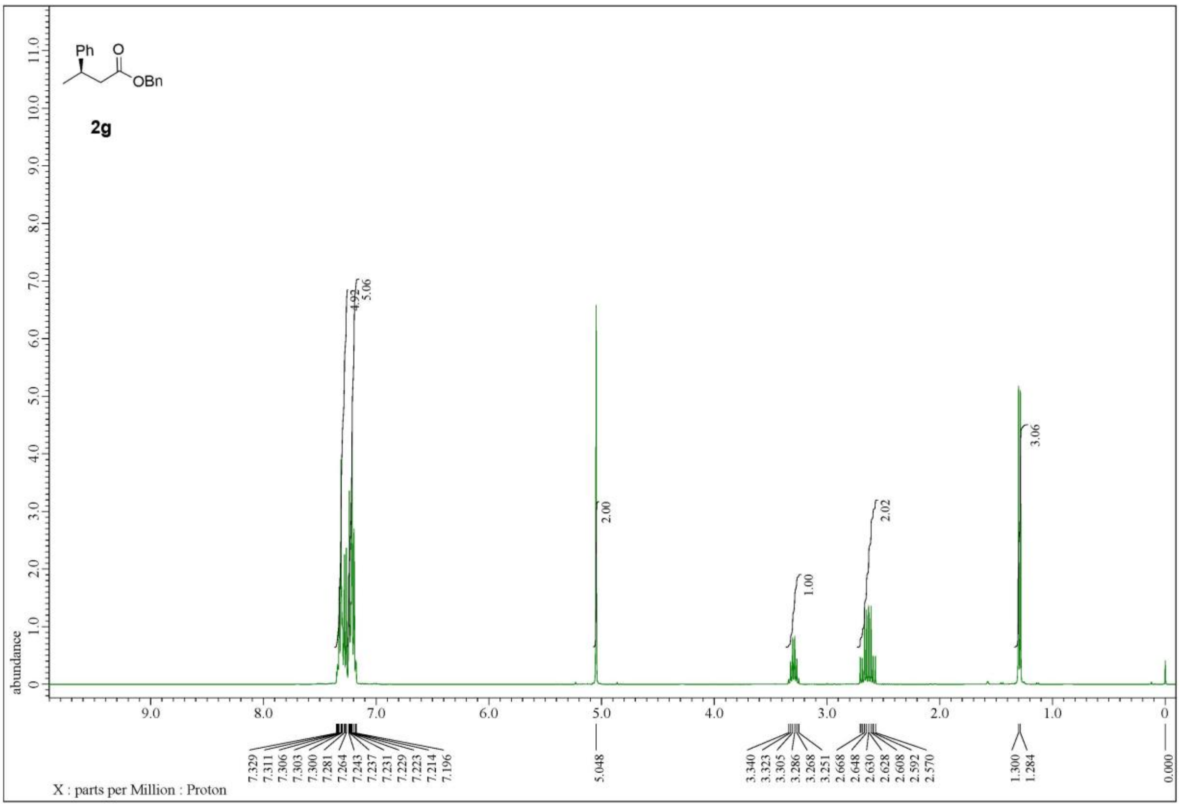
Ph
O
OBn
2g
abundance
X : parts per Million : Proton
7.329
7.311
7.306
7.303
7.300
7.281
7.264
7.243
7.237
7.231
7.229
7.223
7.214
7.196
5.048
3.340
3.323
3.305
3.286
3.268
3.251
2.668
2.648
2.630
2.628
2.608
2.592
2.570
1.300
1.284
0.000
4.92
5.06
2.00
1.00
2.02
3.06

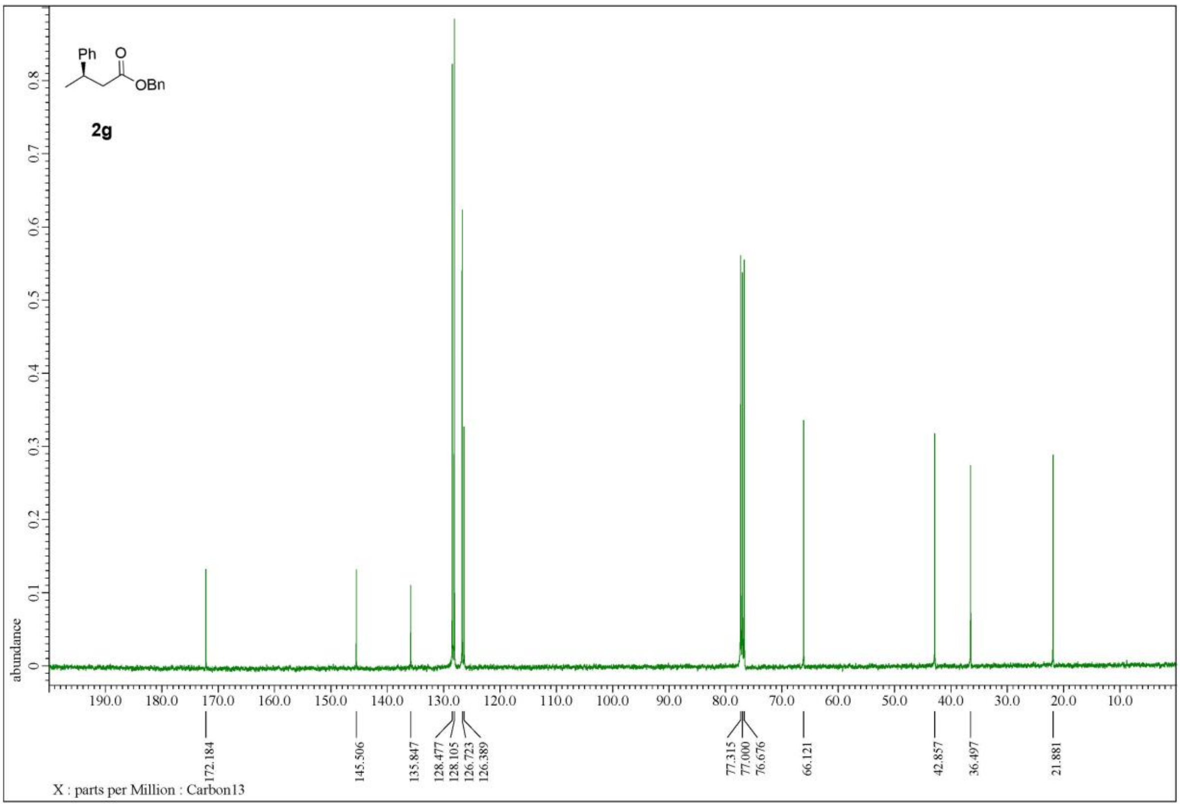
Ph
O
OBn
2g
abundance
X : parts per Million : Carbon13
172.184
145.506
135.847
128.477
128.105
126.723
126.389
77.315
77.000
76.676
66.121
42.857
36.497
21.881

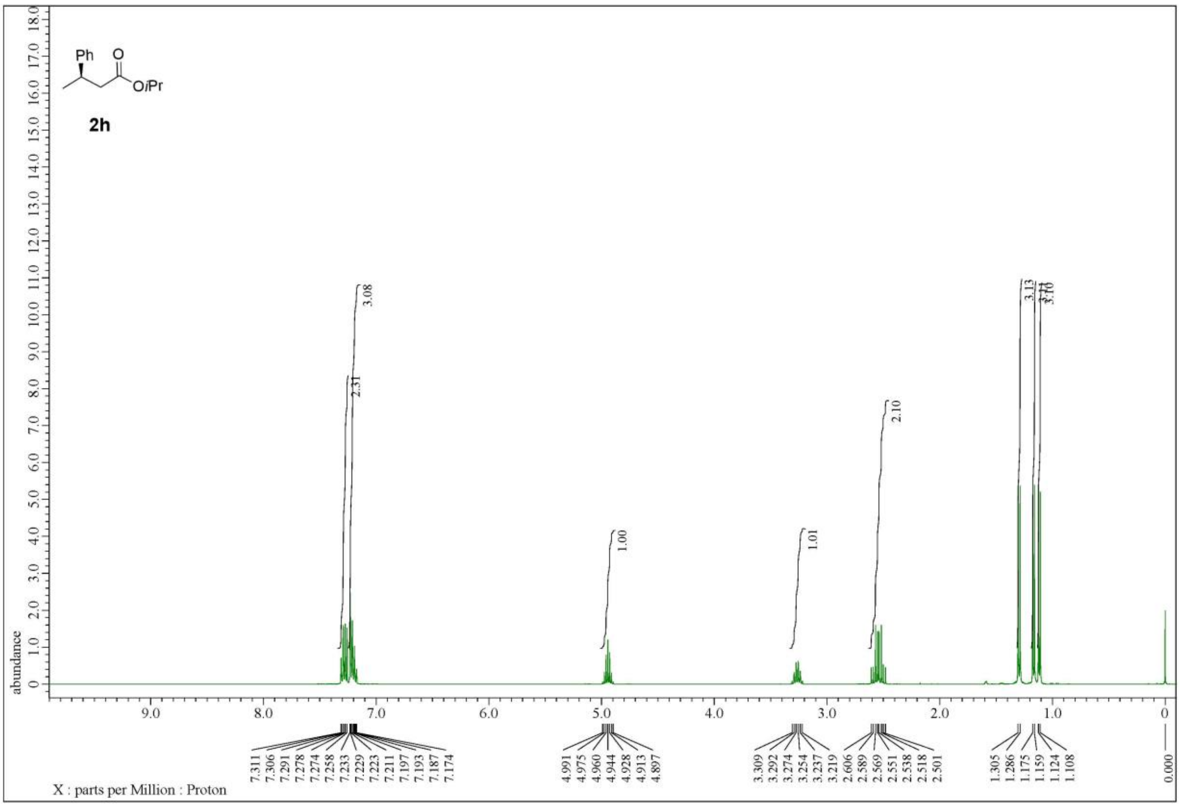
Ph
O
O*i*Pr
2h
X : parts per Million : Proton
abundance

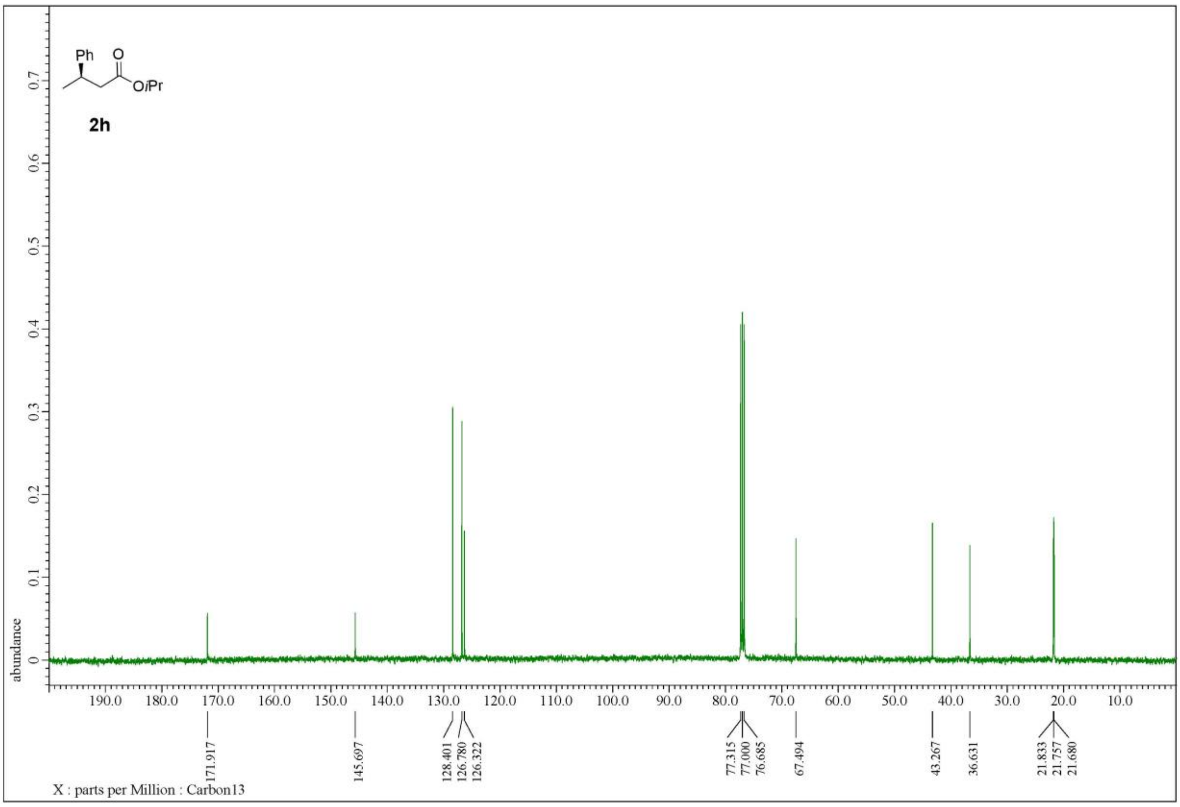
Ph
O
O*i*Pr
2h
X : parts per Million : Carbon13
abundance

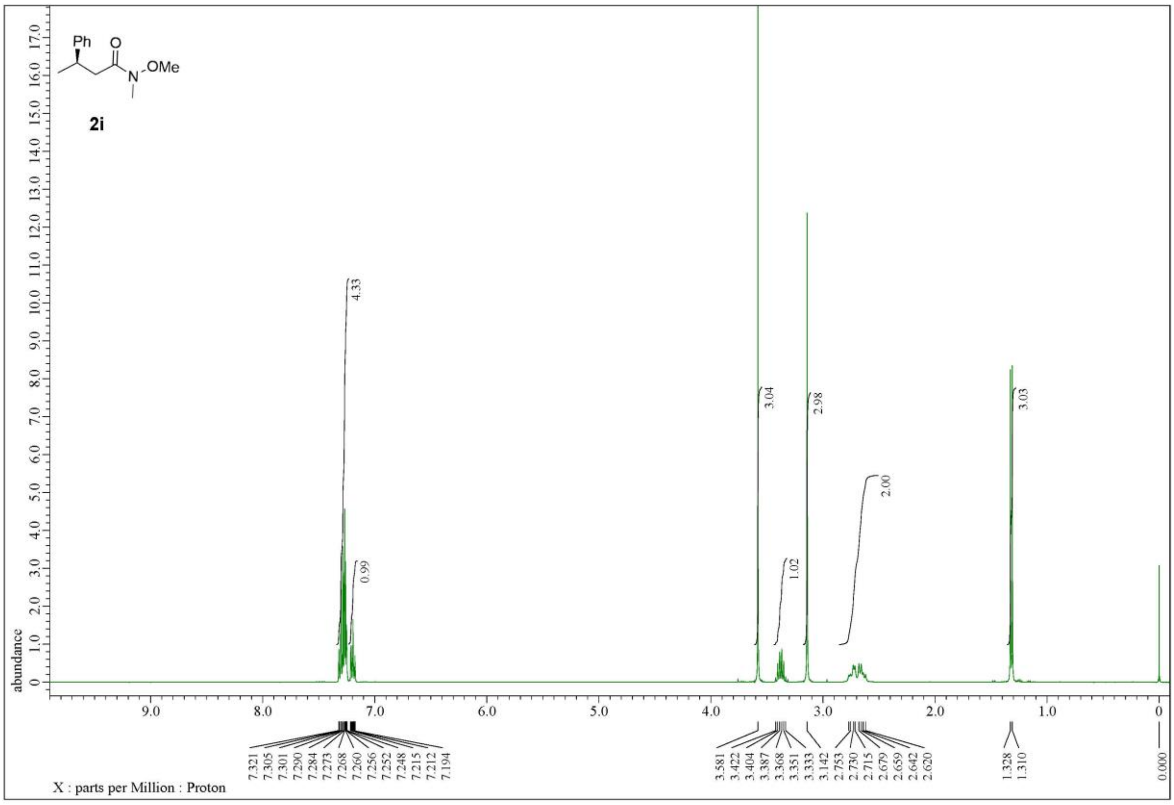

Ph
O
N
OMe
2i
4.33
0.99
3.04
1.02
2.98
2.00
3.03
abundance
X : parts per Million : Proton
7.321
7.305
7.301
7.290
7.284
7.273
7.268
7.260
7.256
7.252
7.248
7.215
7.212
7.194
3.581
3.422
3.404
3.387
3.368
3.351
3.333
3.142
2.753
2.730
2.715
2.679
2.659
2.642
2.620
1.328
1.310
0.000

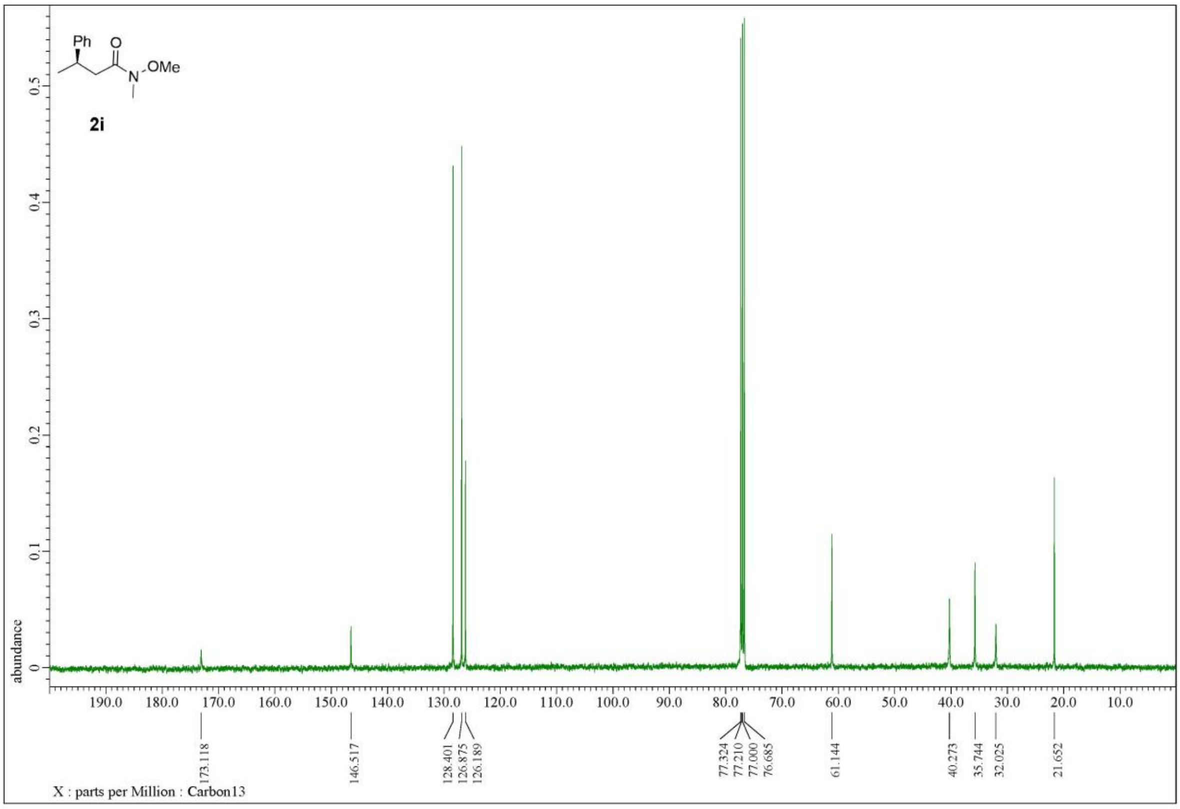

Ph
O
N
OMe
2i
abundance
X : parts per Million : Carbon13
173.118
146.517
128.401
126.875
126.189
77.324
77.210
77.000
76.685
61.144
40.273
35.744
32.025
21.652
